# Physiological basis for xenotransplantation from genetically modified pigs to humans

**DOI:** 10.1152/physrev.00041.2023

**Published:** 2024-03-22

**Authors:** Leigh Peterson, Magdi H. Yacoub, David Ayares, Kazuhiko Yamada, Daniel Eisenson, Bartley P. Griffith, Muhammad M. Mohiuddin, Willard Eyestone, J. Craig Venter, Ryszard T. Smolenski, Martine Rothblatt

**Affiliations:** ^1^ United Therapeutics Corporation, Silver Spring, Maryland, United States; ^2^Imperial College London, London, United Kingdom; ^3^Department of Surgery, Division of Transplantation, Johns Hopkins Medicine, Baltimore, Maryland, United States; ^4^University of Maryland Medical Center, Baltimore, Maryland, United States; ^5^J. Craig Venter Institute, Rockville, Maryland, United States; ^6^Department of Biochemistry, Gdańsk Medical University, Gdańsk, Poland

**Keywords:** genetically modified pigs, xenotransplantation

## Abstract

The collective efforts of scientists over multiple decades have led to advancements in molecular and cellular biology-based technologies including genetic engineering and animal cloning that are now being harnessed to enhance the suitability of pig organs for xenotransplantation into humans. Using organs sourced from pigs with multiple gene deletions and human transgene insertions, investigators have overcome formidable immunological and physiological barriers in pig-to-nonhuman primate (NHP) xenotransplantation and achieved prolonged pig xenograft survival. These studies informed the design of Revivicor’s (Revivicor Inc, Blacksburg, VA) genetically engineered pigs with 10 genetic modifications (10 GE) (including the inactivation of 4 endogenous porcine genes and insertion of 6 human transgenes), whose hearts and kidneys have now been studied in preclinical human xenotransplantation models with brain-dead recipients. Additionally, the first two clinical cases of pig-to-human heart xenotransplantation were recently performed with hearts from this 10 GE pig at the University of Maryland. Although this review focuses on xenotransplantation of hearts and kidneys, multiple organs, tissues, and cell types from genetically engineered pigs will provide much-needed therapeutic interventions in the future.

## Graphical abstract

**Figure:**
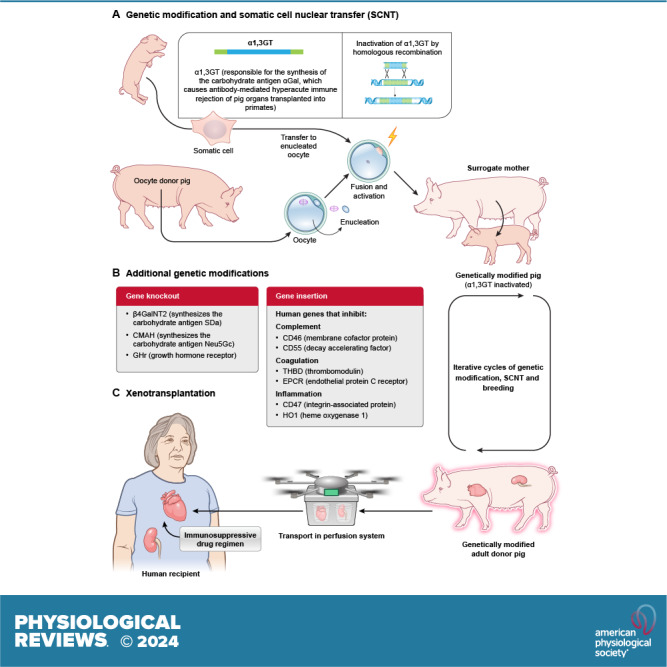

CLINICAL HIGHLIGHTS

- Organ transplantation is the only life-saving intervention for many patients with organ failure.
- Because of a multitude of factors, including the extreme shortage of organ donors, as of February 2024 over 100,000 Americans are on the National Transplant waiting list, with over 6,000 patients dying each year while waiting according to the US Health Resources and Services Administration. Many additional patients not eligible for the waiting list are suffering from end-stage organ failure.
- An unlimited supply of transplantable organs would obviate the need for a multiyear organ transplant waiting list.
- Xenotransplantation of organs from genetically modified, immunologically matched, pathogen-free pigs is one solution to increase the supply of transplantable organs.

Listen to this article’s corresponding podcast at https://physrev.podbean.com/e/xenotransplantation-from-genetically-modified-pigs-to-humans/.

## 1. INTRODUCTION

Living tissues and organs have unique structures, which are complex and elegant and have evolved to fill specific functions. These functions contribute to longevity and quality of life for the individual. Damage to these organs due to a variety of disease processes can progress to become irreversible and require replacement. Although artificial organs can be an answer, biological organs are a potentially more realistic solution for transplantation. The seminal work of Sir Peter Medawar in the middle of the twentieth century showed that the immune system is not an absolute barrier to organ transplantation and that specific immune tolerance can be achieved in mice (1, 2). This resulted in the development of strategies to induce nonspecific immune tolerance, which made human allotransplantation one of the main success stories of that century. However, the massive increase in demand, coupled with the extreme shortage of human donors (3), renewed interest in xenotransplantation, with unique but solvable immunological, surgical, and ethical challenges. This review is an attempt to describe the landscape and the way forward.

## 2. SOURCES OF TRANSPLANTABLE ORGANS

A practical source for xenotransplantation must be not only anatomically and physiologically compatible with humans but also readily accessible and logistically sustainable. Although results from early cases of xenotransplantation between nonhuman primates (NHPs) and humans demonstrated proof of principle, it was evident that NHPs would not provide an acceptable long-term solution for organ procurement (4–8). NHPs share the greatest genetic homology with humans, but they are not readily accessible, produce few offspring, have viruses and other pathogens that can be transmitted to humans, and require several years to reach sexual maturity, and most do not grow adult human-sized organs. Furthermore, ethical, logistical, and scientific concerns make them a poor choice for source animals (9). Pigs, however, exhibit several characteristics that render them a more useful source for human xenotransplantation. Pigs are easily bred in captivity, have a gestation period of ∼4 mo, have relatively large litters of 5–12 offspring, and reach reproductive maturity within 4–8 mo. Most importantly, relative to other large animals pig organs, in particular hearts and kidneys, are anatomically and physiologically similar to human organs (see sect. 5). Since hundreds of millions of pigs are used annually for human consumption, there should be little ethical objection to using pig organs for treating human disease. Pigs can also be raised in designated pathogen-free (DPF) facilities to mitigate the already low risk of zoonotic transmission.

### 2.1. Immunological Barrier to Using Wild-Type Pigs as Human Organ Source

Despite the advantages and anatomical similarities, wild-type pigs are an impractical source for human xenografts. Genetic differences create immunological barriers that separate pigs and humans, as each diverged from a common ancestor >80 million years ago (10, 11). Disappointingly, initial attempts to transplant wild-type pig organs into NHP models failed within minutes to hours of xenograft reperfusion because of hyperacute rejection (HAR) (12, 13). The single known case of wild-type pig-to-human cardiac xenotransplantation was performed in 1997 by Dhani Ram Baruah. Reportedly, within 1 wk the patient died because of fulminant rejection, sparking a major controversy and resulting in the arrests of Dr. Baruah and the surgeon assisting him (14).

**FIGURE 1.**
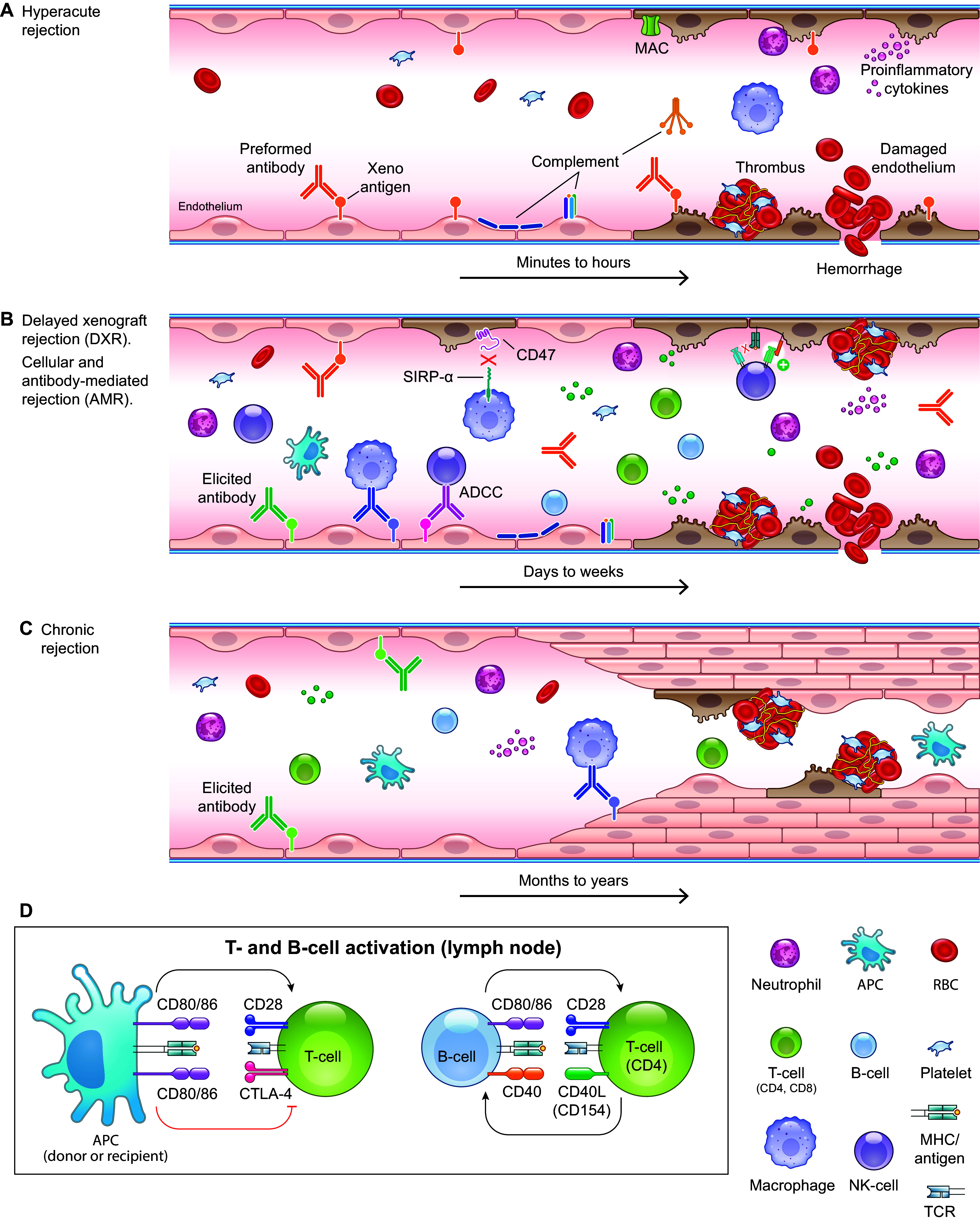
Mechanisms of rejection during xenotransplantation. *A*: hyperacute rejection (HAR). Preformed anti-pig antibodies in the recipient’s blood recognize xenoantigens on vascular endothelial cells in the pig graft and activate complement, leading to endothelial injury, inflammation, interstitial hemorrhage, thrombus formation, and ischemia. *B*: delayed (cellular and antibody mediated) xenograft rejection (DXR) with cellular infiltration and production of elicited antibodies and cytokines. Porcine major histocompatibility complex (MHC) class I [swine leukocyte antigen (SLA I)] molecules may not sufficiently interact with the inhibitory receptors on primate NK cells, leaving the porcine xenograft vulnerable to NK cell cytotoxicity. Porcine CD47 is not sufficient to inhibit primate SIRP-α, which leaves the porcine xenograft susceptible to macrophage phagocytosis. *C*: chronic rejection with chronic inflammation and recurring antibody- and cellular-mediated rejection events within the graft vascular endothelium result in thrombotic microangiopathy, proliferation of endothelial cells, vessel narrowing, and interstitial fibrosis. *D*: T-cell activation by costimulatory signaling provided by T-cell receptor (TCR) recognition of the antigen/MHC and the CD28 interaction with CD80/86; inhibition of T-cell activation via the cytotoxic T lymphocyte-associated protein 4 (CTLA-4)/CD80/86 interaction; B-cell activation by costimulatory signaling provided by TCR recognition of the antigen/MHC class II complex presented by the B cell and the CD40/CD40L (CD154) interaction. ADCC, antibody-dependent cellular cytotoxicity; AMR, antibody-mediated rejection; APC, antigen-presenting cell; MAC, membrane attack complex; NK, natural killer; RBC, red blood cell.

**FIGURE 2.**
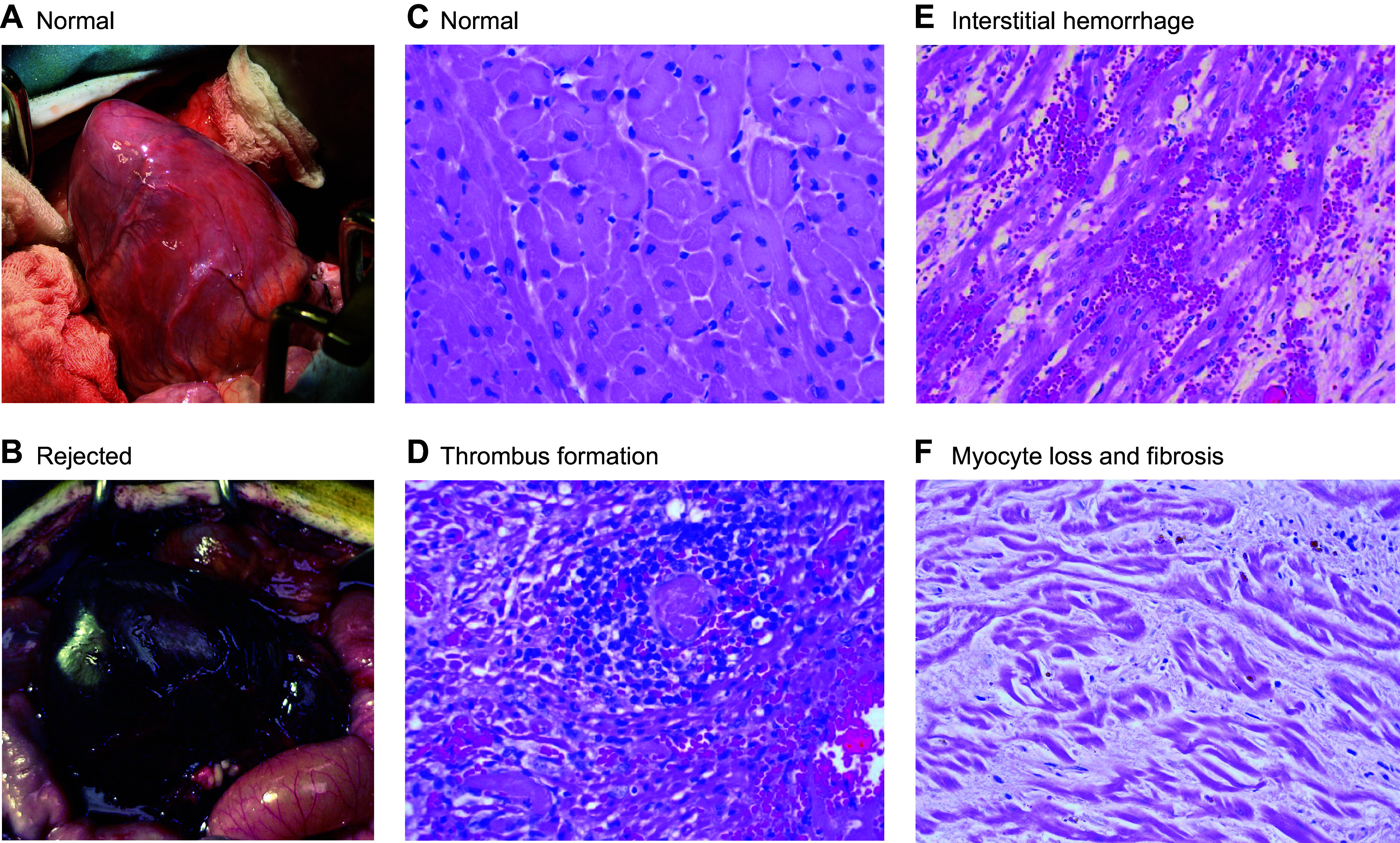
Wild-type pig heart after transplantation into baboon recipient. *A*: whole heart before reperfusion. *B*: whole heart after reperfusion demonstrating hyperacute rejection (HAR). *C*: normal heart tissue before transplant. *D*: heart tissue after transplant demonstrating thrombus formation. *E*: heart tissue after transplant demonstrating interstitial hemorrhage. *F*: heart tissue after transplant demonstrating myocyte loss and fibrosis. Image by Muhammad Mohiuddin.

**FIGURE 3.**
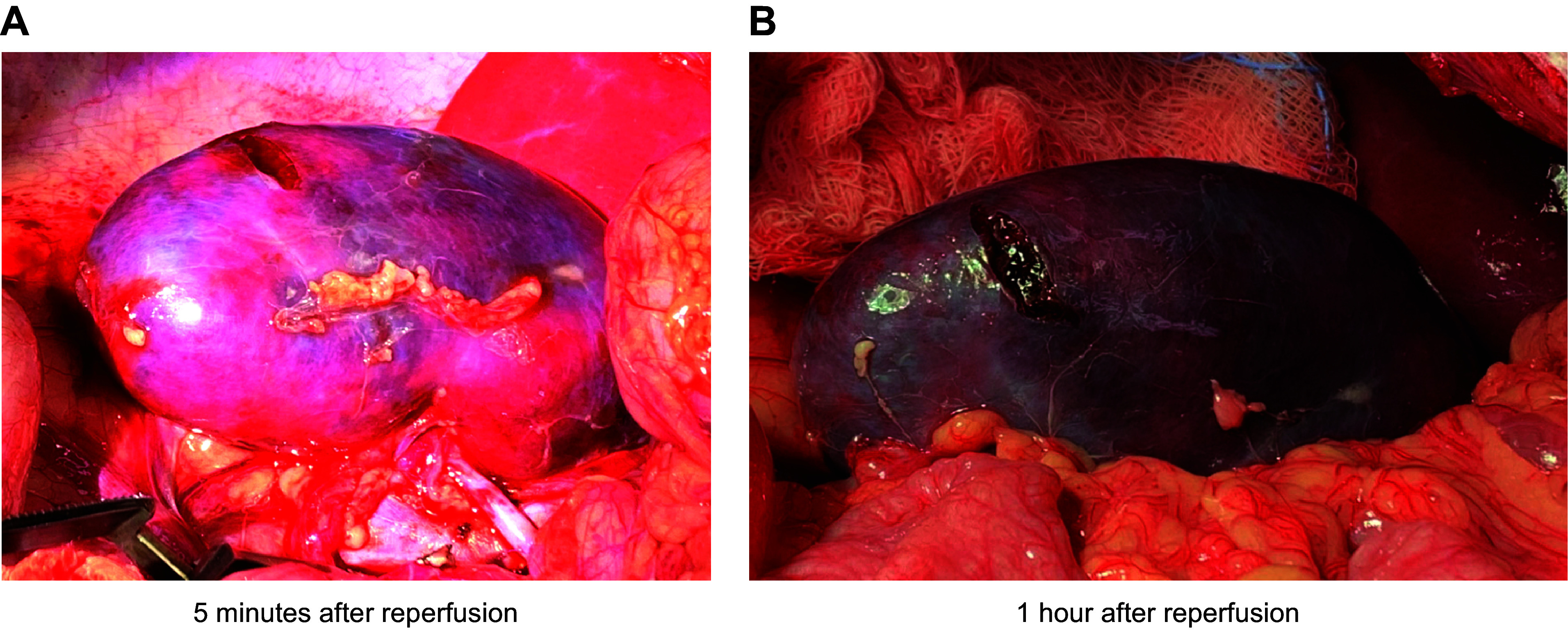
Wild-type pig kidney after transplantation into baboon recipient. *A*: whole kidney 5 min after reperfusion. *B*: whole kidney 1 h after reperfusion demonstrating hyperacute rejection (HAR). Image by Kazuhiko Yamada.

HAR is initiated when preformed anti-pig antibodies in the recipient’s blood recognize xenoantigens on vascular endothelial cells in the pig graft. Antibody-mediated complement activation leads to inflammation, formation of the membrane attack complex (MAC), endothelial injury, and activation of the coagulation cascade. As a result, interstitial hemorrhage, thrombus formation, and ischemia ultimately destroy the graft. Immunohistochemistry analyses of tissues that suffered HAR reveal widespread deposition of immunoglobins and terminal complement products (FIGURE 1, FIGURE 2, AND FIGURE 3) (15, 16).

Membrane-associated complement regulatory proteins (CRPs) expressed on the surface of most cell types negatively regulate complement activation to prevent damage to healthy cells. Coagulation factors also present in the vascular endothelium regulate the coagulation cascade to promote an anticoagulant state under normal conditions. Although porcine-derived complement and coagulation regulatory factors are expressed in the xenograft, they do not efficiently interact with primate-derived (NHP or human) components of the complement and coagulation pathways, resulting in unchecked complement activation and coagulation in the xenograft (17).

The most abundant preformed antibodies in most Old World primates (including humans) recognize the Galα1-3Galβ1-4GlcNAc-R epitope (αGal), which comprise greater than 1% and 4% of total immunoglobulin G (IgG) and immunoglobulin M (IgM), respectively (18, 19). Anti-Gal antibodies are produced in response to chronic exposure to αGal produced by the intestinal microbiome (20). αGal is formed by the enzyme α(1,3)galactosyltransferase (α1,3GT), which catalyzes the transfer of the galactose moiety from UDP-galactose to N-glycan terminal galactose (21). Whereas the gene for α1,3GT is functional in most mammals, it is inactive in humans, apes, and Old World monkeys because of a mutation that occurred after the evolutionary divergence of New and Old World primates (22, 23). The loss of *α1,3GT* gene expression enabled the production of anti-Gal antibodies, which may have provided protection from Old World pathogens that synthesize αGal-like carbohydrate structures, resulting in a selection advantage (19, 22, 24). The role of αGal as the primary epitope responsible for the immune rejection of xenografts was first identified by David Cooper (24).

Initial attempts to deplete preformed anti-pig antibodies in the recipient before pig organ xenotransplantation included conventional plasmapheresis to remove serum proteins, immunoadsorption columns to remove IgG and IgM antibody fractions or anti-Gal antibodies specifically (25), perfusing recipient plasma or whole blood through a separate “sponge” pig organ to absorb anti-pig antibodies, and bathing pig donor organs in α-galactosidase to remove the αGal epitope. Additionally, decoy carbohydrate polymers (Gal glycoconjugates) were administered to the recipient to compete with αGal on the xenograft for anti-Gal antibody binding (26). Although these techniques delayed HAR, anti-Gal antibodies eventually returned to circulation and triggered xenograft rejection. Additional approaches to prevent HAR in pig-to-baboon transplants involved the administration of agents that deplete or inhibit complement such as cobra venom factor (CVF) or soluble complement receptor-1 (sCR-1) (27–31). As was true for anti-Gal antibody depletion techniques, HAR was delayed but eventually the pig xenograft was rejected.

In the rare cases where HAR did not occur, delayed xenograft rejection (DXR) or acute vascular rejection, which occurs within days to weeks, would lead to organ failure (FIGURE 1). DXR includes both cellular and antibody-mediated rejection (AMR). Cellular xenograft rejection involves the innate and adaptive immune systems, primarily natural killer (NK) cells, macrophages, and T cells (32). NK cytotoxicity can occur through direct interaction of NK cells with donor endothelial cells or by indirect NK interaction via NK cell recognition of antibody bound to target antigen. The latter process, termed antibody-dependent cellular cytotoxicity (ADCC), involves the binding of FcγRIII (CD16) on the NK cell to the Fc domain of preformed or elicited antibodies, leading to NK cell activation and release of cytotoxic factors. NK cells express inhibitory receptors that upon detection of major histocompatibility complex (MHC) class I molecules negatively regulate NK activating receptors to prevent killing of normal healthy cells. However, porcine MHC class I [swine leukocyte antigen (SLA I)]molecules may not sufficiently interact with the inhibitory receptors on primate NK cells, thereby leaving the porcine xenograft vulnerable to NK cell cytotoxicity. Macrophages also participate in rejection by phagocytosis [mediated by scavenger receptors, Fc receptors, and complement receptors (33)] and by producing proinflammatory cytokines. Although normal healthy cells express CD47, which interacts with signal regulatory protein alpha (SIRP-α) on macrophages and transmits a “don’t eat me” signal, porcine CD47 is not sufficient to inhibit primate SIRP-α, leaving the porcine xenograft susceptible to macrophage phagocytosis.

T cells play the most prominent role in DXR. T-cell activation, proliferation, and differentiation require the engagement of the T-cell receptor (TCR) with the peptide antigen/MHC complex on the antigen-presenting cell (APC), as well as an additional interaction referred to as costimulation. Multiple costimulatory pathways have been identified, but the most understood involves an interaction between CD28 on the T cell and CD80/86 on the APC (FIGURE 1). T-cell activation by the APC may occur by direct, indirect, or semidirect mechanisms. The direct mechanism involves activation of recipient T cells by donor APCs (i.e., passenger APCs transplanted along with the xenograft). In this case, the donor APC (with swine antigen/swine MHC complex) migrates to the lymph node and directly activates recipient T cells with the appropriate TCR. Because swine MHC (SLA) is ∼70% homologous to human MHC [human leukocyte antigen (HLA)], a subset of the swine antigen/swine MHC complexes is recognized by primate TCRs. In contrast, the indirect pathway for T-cell activation involves the uptake and processing of xenoantigens by recipient APCs and presentation to T cells by recipient MHC molecules. Finally, the semidirect mechanism involves trogocytosis of the entire swine antigen/swine MHC complex from a donor cell by a recipient APC. The recipient APC then presents the swine antigen/swine MHC complex to recipient T cells. Once activated, T cells travel to the graft where cytotoxic (CD8) T cells attack directly and helper (CD4) T cells promote other cell responses through receptor-ligand interactions (e.g., B cells) or secreted cytokines and chemokines (e.g., NK cells, macrophages, neutrophils, cytotoxic T cells). Cell-mediated rejection is characterized histologically by leukocyte infiltration, with individual cell types identified by immunochemistry. Cytotoxic T lymphocyte-associated protein 4 (CTLA-4) is also expressed by T cells and binds CD80/CD86 on APCs. However, this interaction inhibits T-cell activation and unwanted action against self-tissues (FIGURE 1). Upon CTLA-4 binding, CD80 and CD86 undergo transendocytosis and degradation by the T cell, rendering them inaccessible for CD28 costimulation (34). A fusion protein consisting of a modified extracellular domain of CTLA-4 and a portion of the Fc domain of human IgG1 (CTLA-4 Ig; belatacept), which binds CD80 and CD86 and blocks the CD28 T-cell costimulation pathway, has been developed for prophylaxis of kidney rejection after transplant (NULOJIX USPI).

Antibody-mediated rejection (AMR) involves the production of elicited antibodies by activated B cells. B-cell activation is initiated by the binding of the B-cell receptor to an antigen, which can occur independently of T cells; however, affinity maturation, isotype switching, and production of plasma and memory B cells require help from activated T cells. This activation requires two signals: one is through T-cell receptor recognition of the xenopeptide/MHC class II complex presented by the B cell, and the other is a costimulatory signal provided through the CD40 receptor binding its ligand (CD40L or CD154) on T cells (FIGURE 1). Blocking the CD40L-CD40 interaction with anti-CD40 or anti-CD154 monoclonal antibodies (MAbs) results in the inhibition of B-cell expansion and decreased antibody production. The histological characteristics of AMR are similar to those seen with HAR and include inflammation, interstitial hemorrhage, infarction, thrombosis, necrosis, and deposition of immunoglobins, complement deposition, fibrin, and platelets.

Chronic rejection, which has only been observed in the years after modification of donor pigs as wild-type pigs were all rejected within days to weeks, can occur months or years after transplant and is believed to involve chronic inflammation and recurring antibody- and cellular-mediated rejection events within the graft vascular endothelium (FIGURE 1). It is characterized by thrombotic microangiopathy (TMA), proliferation of graft vascular endothelial cells, vessel narrowing, and interstitial fibrosis, ultimately causing hypoperfusion of the graft and failure.

Given the immunological barriers associated with transplantation of organs from wild-type pigs, which uniformly resulted in early graft rejection and failure, it became clear that long-term function of pig xenografts in humans required genetics-based technological advancements such that specific genes within the pig could be inactivated and human genes introduced.

The technological advancements discussed in the following sections have been made possible through the collective efforts of generations of scientists, including several Nobel laureates, over multiple decades of discoveries and developments. Cloning and genetic engineering technologies can now be used to further enhance the suitability of pig organs for xenotransplantation into humans.

## 3. SCIENTIFIC AND TECHNOLOGICAL ADVANCES THAT ACCELERATED/INFORMED XENOTRANSPLANTATION

### 3.1. Editing the Mammalian Genome

Homologous recombination is a fundamental process utilized by all life forms whereby nucleotides are exchanged between two similar sequences of DNA to facilitate DNA repair, DNA replication, and genetic diversity. Mammalian genome editing, or gene targeting, is the ability to harness the homologous recombination machinery within any living cell to precisely alter its DNA sequence (35). Gene targeting is one of the most powerful techniques used in basic science and biomedical research. It has been used to create mutations in >8,000 mouse genes and generate >1,000 human disease models (36). Without gene targeting, successful pig-to-human xenotransplantation would not be possible.

For discovering and developing the principles of gene targeting, the 2007 Nobel Prize in Physiology or Medicine was awarded to Mario Capecchi, Martin Evans, and Oliver Smithies (37). Several features have been added to the technology over the years to improve efficiency or to direct the genetic modification to a specific tissue or developmental stage, but the fundamental mechanism of homologous recombination discovered ∼40 years ago is still the basis of mammalian genome editing used today (38–41). Gene targeting was initially used to inactivate endogenous genes (i.e., to “knock out” genes) (42, 43). It has since been used to insert exogenous DNA sequences into specific locations in the genome (targeted insertion or “knockin”), replace specific DNA sequences, and generate single-nucleotide point mutations. The most elaborate genetically engineered (GE) pigs currently used for xenotransplantation have been produced by using highly innovative approaches to incorporate multiple gene knockouts (KOs) and targeted insertions.

Mario Capecchi’s desire to understand the genetic basis of mammalian development by systematic gene inactivation and phenotype analysis (reverse genetics) led him to begin working on techniques in the mid-1970s to achieve gene targeting in mice. It had been demonstrated that, under certain conditions, DNA fragments containing a specific gene could be added to cultured mammalian cells deficient in that gene, stably incorporate into the genome, and restore the deficient phenotype at a rate of ∼1 per 1,000,000 cells (44). To improve efficiency, Capecchi designed glass micropipettes to inject DNA directly into the nuclei. The technique was effective, such that a functional gene integrated into the genome of 1 in 3 cells (45). Soon thereafter, DNA microinjection was used by several groups to generate the first transgenic mice (46–49). For the next two decades, microinjection of DNA was the primary method used to generate genetically altered animals, including transgenic pigs, rabbits, sheep, and cattle for agricultural purposes and medical research (50, 51).

#### 3.1.1. Generation of transgenic animals.

**FIGURE 4.**
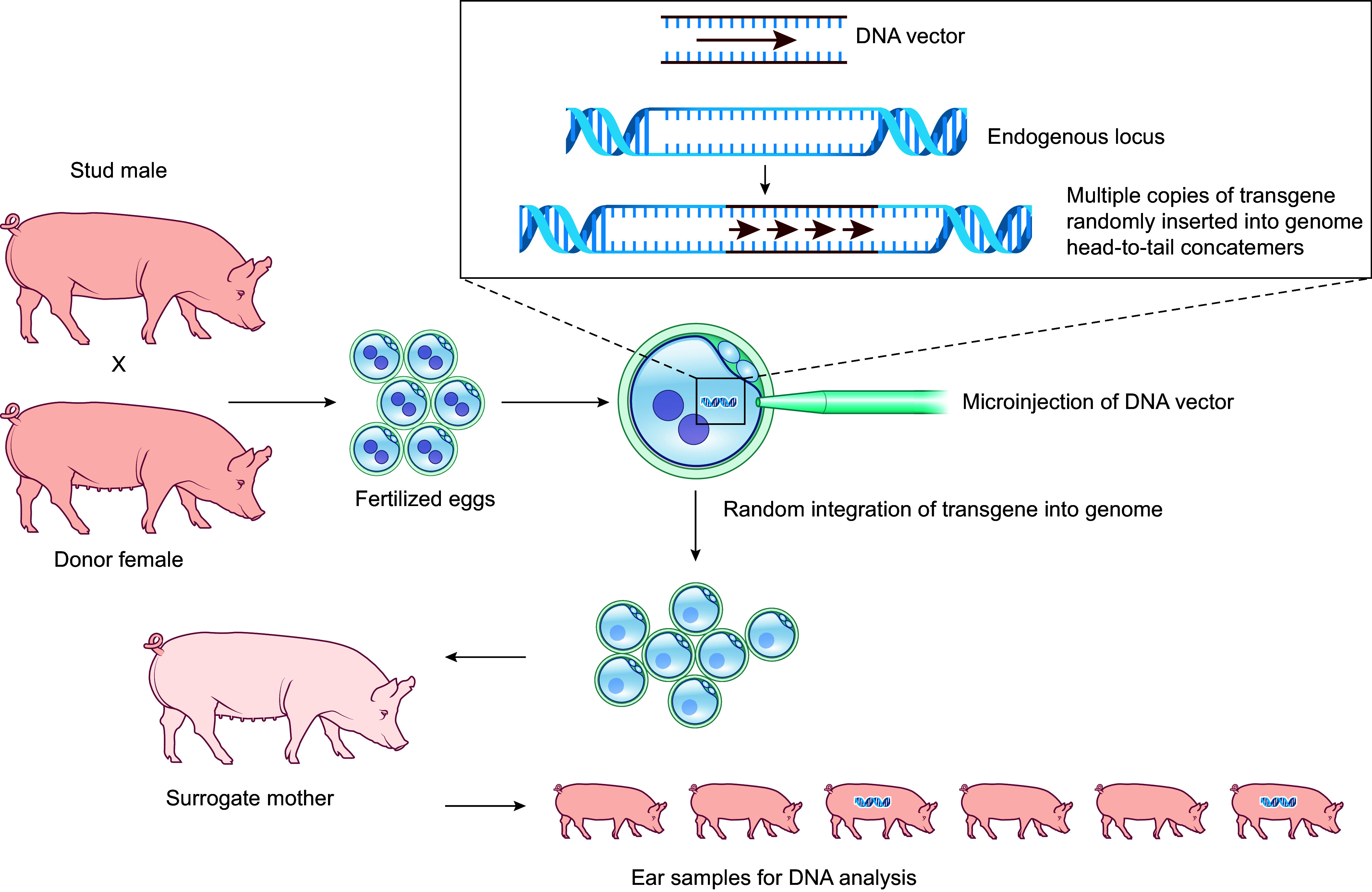
Generation of transgenic pigs. Transgenic pigs are produced by injecting a DNA vector containing the transgene and a promoter into the pronuclei of fertilized eggs. The transgene integrates into the genome as head-to-tail DNA concatemers as a result of homologous recombination. Embryos are transferred into the oviduct of a surrogate mother to complete gestation. Approximately 10% of live births result in transgenic animals.

Transgenic animals are produced by injecting an exogenous DNA vector (containing a gene of interest plus a regulatory promoter to direct its expression) into the nuclei of single-cell embryos. Surviving embryos are transferred into the oviduct of a surrogate mother to complete gestation (FIGURE 4). Although some or all cells of each resulting animal may have one or more copies of the transgene integrated into the genome, the location varies between animals since transgenes randomly integrate. A limitation of random integration is that the transgene may disrupt or cause aberrant expression of endogenous genes, resulting in confounding and potentially detrimental phenotypes. In addition, randomly integrated transgenes may integrate into a quiescent region of the genome and be expressed at very low levels, or not at all. Nevertheless, by the mid-1990s transgenic pigs for human CRPs (e.g., CD55, CD59, and CD46) were generated for xenotransplantation (52–54), and several xenotransplantation companies (e.g., Imutran, Alexion, XenoTrans) were founded based on this technology (discussed in sect. 4.1).

#### 3.1.2. Gene targeting using homologous recombination in embryonic stem cells.

Capecchi’s group noted that before integration into the genome, copies of the transgene undergo homologous recombination to form head-to-tail DNA concatemers (38). If homologous recombination occurred between exogenous DNA fragments, Capecchi speculated that homologous recombination would also occur between injected engineered DNA fragments (or “targeting vectors”) and the homologous counterpart in the genome, thus enabling predefined genetic edits of any endogenous locus.

During the same time frame, Oliver Smithies was also well aware of the power of homologous recombination in gene therapy applications. Smithies collaborated with the laboratory of Raju Kucherlapati and demonstrated that, as in prokaryotic systems, a double-strand break (DSB) in the mammalian genome was a critical step in the initiation of strand exchange in homologous recombination and that DSBs occurring within or adjacent to the region of homology significantly increased the frequency of homologous recombination (39, 55).

**FIGURE 5.**
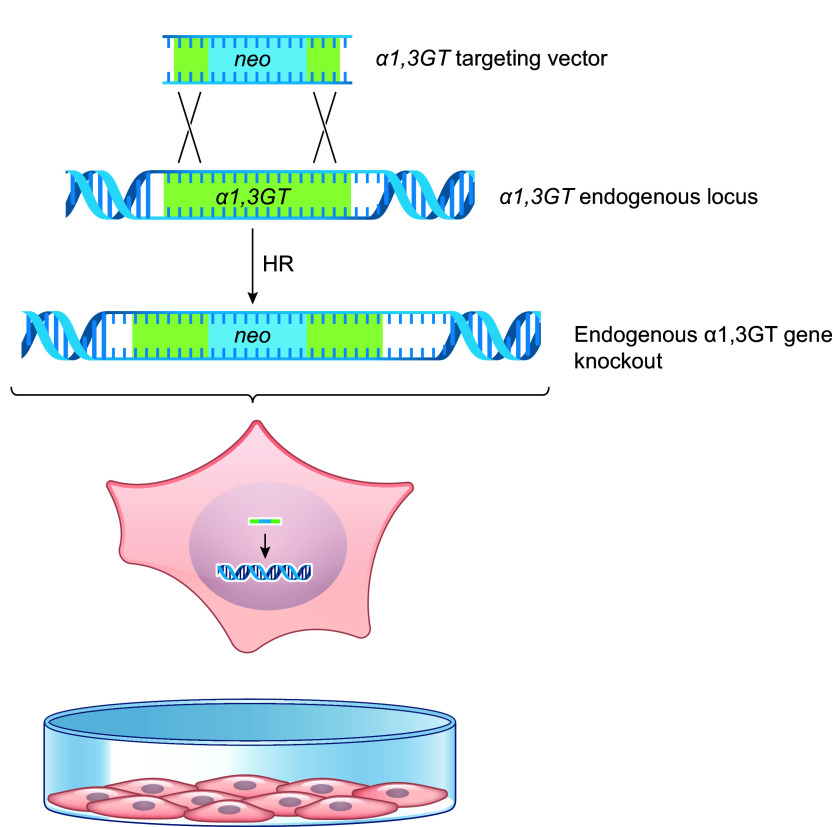
Targeted disruption of the *α1,3GT* gene locus in cultured cells. A targeting vector that includes a portion of the target gene plus adjacent 5′ and 3′ sequences (“homology arms”) with the neomycin resistance gene (*neoR*) inserted into the coding sequence to disrupt the gene. Electroporation to facilitate entry of the DNA into the cell and homologous recombination (HR) with the endogenous DNA results in cells with a heterozygous knockout of the endogenous *α1,3GT* gene and resistance to the antibiotic G418 (a neomycin analog).

By the mid-1980s, both Capecchi and Smithies were independently demonstrating homologous recombination-mediated gene targeting in cultured mammalian cells (40, 41, 56). The targeting vector was constructed using a fragment of DNA from the cells to be modified, including a portion of the target gene plus adjacent 5′ and 3′ sequences (“homology arms”). A functional neomycin resistance gene (*neoR*) was inserted to disrupt the coding sequence of the target gene. Homologous recombination between the targeting vector’s homology arms and cognate endogenous DNA would result in cells with a heterozygous knockout of the endogenous gene and resistance to the antibiotic G418 (a neomycin analog). This original targeting vector design was used to inactivate the porcine *α1,3GT* gene for xenotransplantation (FIGURE 5) (57).

The next challenge was to generate an entire animal from the genetically altered cells. Martin Evans had identified a line of mouse embryonic stem (ES) cells that when injected into a wild-type host embryonic blastocyst would contribute to the germ line of the resulting chimeric mouse (58, 59). Genetic modifications introduced into these ES cells before blastocyst injection could be transmitted through the germ line of the chimera to its F_1_ progeny. Crossing F_1_ heterozygotes would produce a homozygous gene knockout in one-quarter of the offspring (assuming that complete gene inactivation does not result in embryonic lethality) (60–62).

Several laboratories soon acquired the ability to generate homozygous knockout mice; however, gene targeting in other mammals, including pigs and other livestock, was not yet possible, as equivalent ES cells (those capable of contributing to the germ line after injection into a host blastocyst) had not been established in other species. The lack of totipotent ES cells in pigs limited the xenotransplantation field to transgene insertion by microinjection for almost a decade. However, the advent of animal cloning using somatic cell nuclear transfer (SCNT) into enucleated oocytes several years later facilitated gene targeting in farm animals as well (63).

### 3.2. Somatic Cell Nuclear Transfer (Animal Cloning)

Animal cloning is defined as the process of generating a new organism from a cell or cells of a donor organism, whereby the new organism is genetically identical to the donor. Nuclear transfer research ongoing as early as the 1950s in frogs demonstrated that early embryonic (blastula and gastrula stage) nuclei (64, 65) and somatic nuclei from tadpoles (66, 67) transferred into enucleated oocytes could develop into adult frogs. This work established a fundamental principle in biology, which is that spatial and temporal changes to gene expression, rather than permanent changes in the genome, are responsible for the differentiation of most cell types. The discovery that differentiated cells can be reprogrammed to a totipotent state won John Gurdon and Shinya Yamanaka the Nobel Prize in Physiology or Medicine in 2012. Cloning in mammals via nuclear transfer proved to be more difficult than that in frogs because of the small size of the mammalian egg (<0.1% that of an amphibian egg) and the presence of large amounts of lipid in mammalian eggs (especially pig, sheep, cow, and goat) that made it difficult to visualize the nucleus.

The SCNT methodology involves removing the metaphase II chromosomes from mature oocytes and placing a donor cell under the zona pellucida next to each enucleated oocyte. Application of an electric current serves to fuse the cytoplast of the donor cell and oocyte and activates cell division. At this point, the nuclear membrane breaks down to allow ooplasmic “reprogramming factors” access to the chromosomes, which undergo significant epigenetic modifications and remodeling resulting in transition to a state of totipotency (68). After fusion is confirmed, a second electrical pulse is applied to activate development. Reconstructed embryos are then injected into the oviduct of a surrogate mother to complete gestation (69). Three decades after the successful cloning of frogs by nuclear transfer, McGrath and Solter (70) generated cloned mice from donor nuclei of single-celled mouse zygotes.

Throughout the 1980s and 1990s, several groups generated mammalian clones using donor nuclei from undifferentiated early-stage sheep, rabbit, pig, mouse, cow, and monkey embryos (71–76), but since these did not employ cultured diploid cells, genetic modification (i.e., gene knockout) was not possible with this methodology. However, the cloning of two lambs (“Megan” and “Morag”) using nuclei from an established differentiated cell line from a day 9 sheep embryo paved the way for the cloning of “Dolly” the sheep a year later (77) and the ability to genetically manipulate large mammals.

The cloning of Dolly the sheep by the Roslin Institute and PPL Therapeutics was a major scientific breakthrough. As she was cloned from the nucleus of an adult somatic cell (from her donor’s mammary gland), her birth and 6.5-yr life demonstrated for the first time that the genome of an adult differentiated mammalian cell can be reprogrammed to totipotency and generate an entire genetically identical animal (63). The cloning of pigs from cultured adult somatic cells (69) was accomplished by the United States subsidiary of PPL Therapeutics (now Revivicor/United Therapeutics).

**FIGURE 6.**
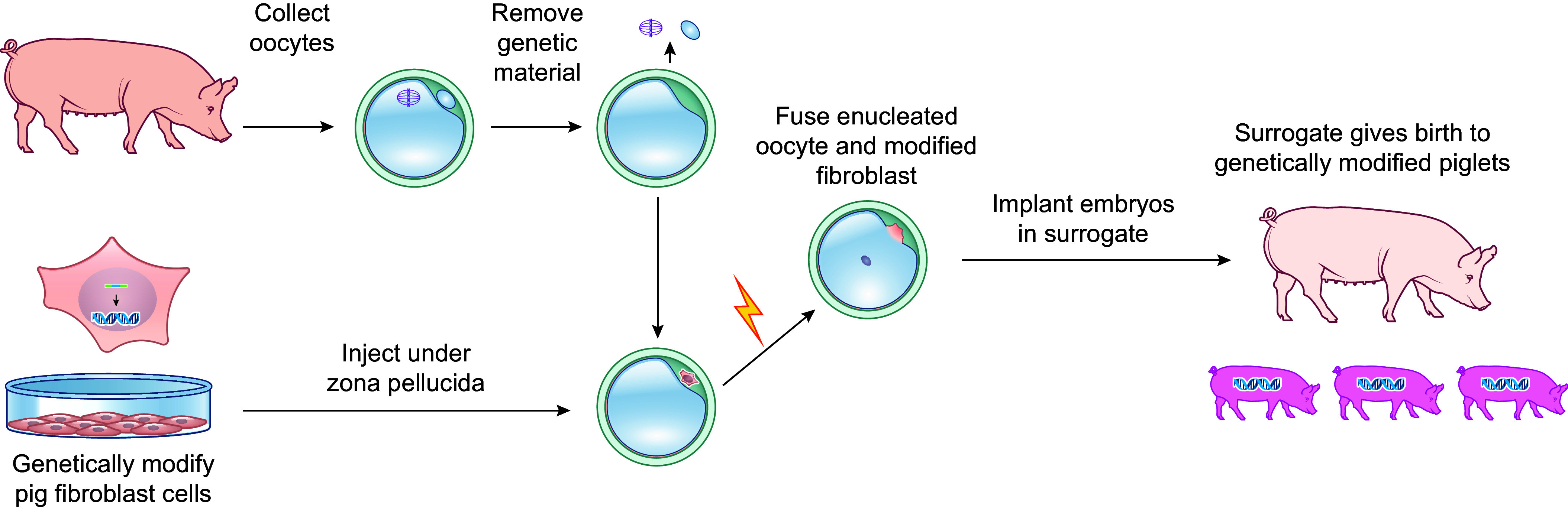
Genetically altering pigs with gene targeting and somatic cell nuclear transfer. The somatic cell nuclear transfer (SCNT) methodology involves removing the metaphase II chromosomes from mature oocytes and placing a donor cell under the zona pellucida next to each enucleated oocyte. Application of an electric current fuses the cytoplast of the donor cell and oocyte and activates cell division. Reconstructed embryos are injected into the oviduct of a surrogate mother to complete gestation.

The ability to clone pigs from cultured somatic cells fundamentally progressed the field of xenotransplantation. Once pig embryos could be reconstructed directly with cultured somatic cells and enucleated oocytes, genetic alterations could be achieved without the need for ES cells or host blastocysts. Gene targeting via homologous recombination in cultured somatic cells followed by nuclear transfer became a viable approach to generate any heritable genetic alteration desired (FIGURE 6).

Although cloning is an essential tool for generating genetically altered pigs, its efficiency is quite low. Less than 5% of cloned embryos develop normally and result in live births (78). Several factors, including donor cell type, oocyte maturation stage, and embryo activation method, all likely contribute to the developmental competence of cloned embryos, although the most likely cause of frequent abortion, developmental abnormalities, and mortality of cloned animals is the inability or delay in resetting the totipotent epigenetic program of the donor nuclei (79).

The epigenetic program influences gene expression in the developing embryo and throughout the animal’s life span and involves DNA methylation, histone modification, and noncoding ribonucleic acid (RNA), all of which impact accessibility of the DNA to transcription factors and enzymes but do not alter the DNA sequence (80). The epigenetic program permits totipotency of the cells of the early embryo. During development, epigenetic modifications to the DNA change to allow for appropriate gene expression and normal cell differentiation. Importantly, for proper embryonic development of the next generation, the epigenetic pattern that allows for totipotency must be restored, which mostly happens during development of the primordial germ line and upon fertilization. Differentiated somatic cells used for cloning bypass germ line specification and fertilization stages and instead move directly into embryogenesis. Therefore, the epigenetic program may not be reset appropriately in clones, resulting in abnormal gene expression during embryonic development and mortality and congenital abnormalities (79). Fortunately, once clones undergo natural breeding, the proper epigenetic program is restored for subsequent generations, and the birth rate returns to normal (69).

### 3.3. High-Efficiency Gene Editing

For two decades classical gene targeting dominated the world of mammalian genetics, with its main limitation being the relative low frequency of the homologous recombination event. Once it was demonstrated that double-strand breaks in mammalian DNA undergo efficient repair by either nonhomologous end joining (NHEJ) or homology-directed repair (HDR) (81, 82), and could thereby facilitate gene targeting, several groups sought to discover rare-cutting, site-specific endonucleases. Initially, zinc finger nucleases were engineered to recognize and cleave unique DNA recognition sites within the mammalian genome; however, these were expensive and time consuming to produce and suffered widespread off-target cleavage (83). Next, transcription activator-like effector nucleases (TALENS) were engineered to include customizable site-specific DNA binding domains (84). Although TALENS are versatile and highly specific, production of the unique DNA binding domains also required labor-intensive protein synthesis and assembly. As such, once the RNA-guided clustered regularly interspaced short palindromic repeat (CRISPR)/CRISPR-associated (Cas) nuclease system was available, it replaced the protein-based systems (85–90).

**FIGURE 7.**
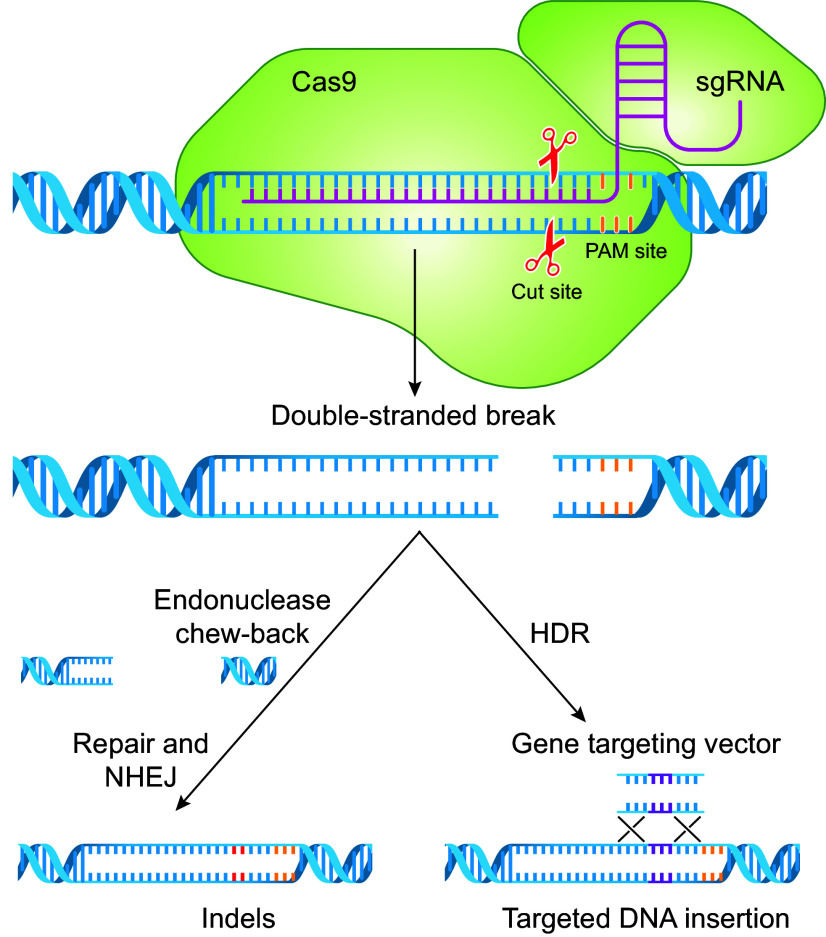
Clustered regularly interspaced short palindromic repeats (CRISPR)-facilitated gene editing. The CRISPR/CRISPR associated (Cas) system consists of a guide RNA with homology to the endogenous target site, a nuclease-binding domain, and a Cas nuclease. The guide RNA directs the Cas nuclease to the target genomic site, where it creates a double-strand break (DSB). Cellular machinery repairs the DSB, which involves the deletion and/or insertion of several nucleotides (indels) and nonhomologous end joining (NHEJ). Indels that occur within an exon can result in a frameshift, leading to generation of a stop codon and translation of truncated, nonfunctional protein. Gene-targeting vectors are used to generate insertions and point mutations at prespecified locations in the genome via homology-directed repair (HDR). PAM, protospacer adjacent motif; sgRNA, single guide RNA.

CRISPR/Cas technology is based on a naturally occurring antiviral mechanism in bacteria and has been cleverly adapted for editing mammalian genomes (91, 92). In 2020, Emmanuelle Charpentier and Jennifer Doudna were awarded the Nobel Prize in Chemistry for the discovery of the CRISPR/Cas9 system (93). The CRISPR/Cas system creates DNA DSBs at prespecified locations in the genome, which initiates cellular DNA repair processes. In its simplest form, the CRISPR/Cas system consists of an RNA sequence with homology to a genomic target at the site of the desired modification (“guide” RNA), a nuclease-binding domain, and a Cas nuclease (FIGURE 7). The guide RNA directs the Cas nuclease to the target genomic site, where it creates a DSB. The cellular machinery repairs this DSB, which typically results in the deletion and/or insertion of several nucleotides (indels) followed by NHEJ. Indels that occur within an exon can result in a frameshift, leading to generation of a stop codon and translation of truncated, nonfunctional protein. Mutagenesis via CRISPR/Cas9 involves transfecting cells with expression vectors containing guide RNA and the Cas nuclease sequences (91) or with ribonucleoprotein particles composed of guide RNA complexed to Cas9 protein.

CRISPR/Cas is also used in combination with gene targeting vectors to generate targeted insertions or to create point mutations at prespecified locations in the genome via HDR (FIGURE 7). Like classical gene targeting, the targeting vector contains the desired genetic alteration flanked by homology arms. A CRISPR/Cas9-mediated DSB at the target location significantly increases the efficiency of the homologous recombination event relative to classical gene targeting 1,000-fold. In some cases, the efficiency is so high that both alleles of the target locus are edited in one step, resulting in a homozygous genomic modification.

Although each guide RNA of the CRISPR complex is designed to anneal to a single location in the genome, it may unexpectedly anneal to additional similar locations elsewhere, resulting in off-target DSBs and unintended mutations. Additionally, the HDR vector may integrate into the genome randomly as a transgene. DNA sequencing is used to rule out off-target mutations, whereas Southern blot and digital drop polymerase chain reaction (PCR) are used to detect random integrations of the HDR vector. Because of the Human Genome Project, the entire genome of the animal can be sequenced efficiently and at low cost to verify genomic identity.

**FIGURE 8.**
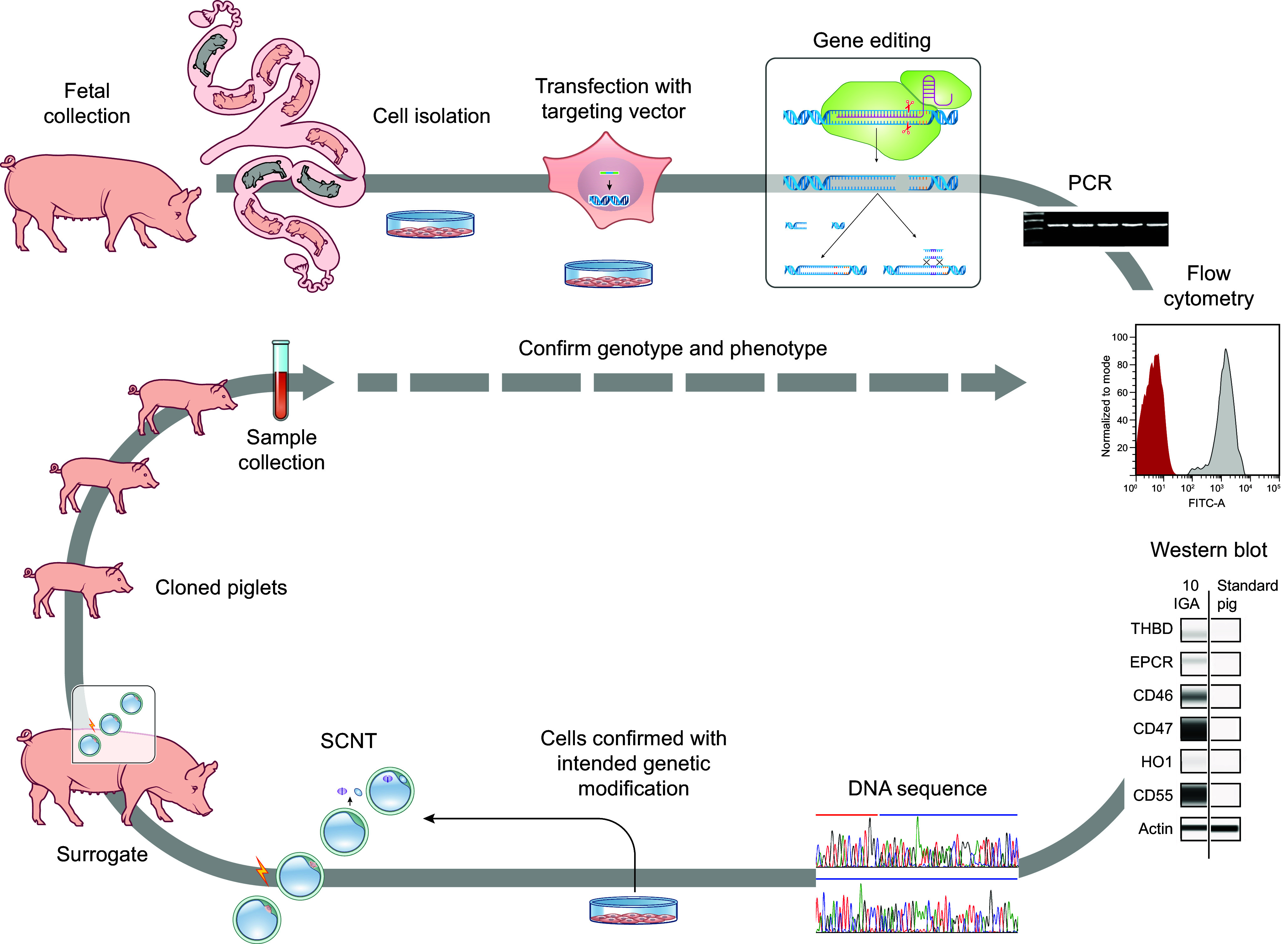
Generation of multiple genetic modifications in the pig. Fetal fibroblasts are collected from midgestation fetuses, cultured, and transfected with a targeting vector for gene editing. Cells are screened for the intended genetic modification and appropriate protein expression (or lack thereof) by polymerase chain reaction (PCR) and DNA sequencing and Western blot and flow cytometry, respectively. Cells with the intended genotype and phenotype are used for somatic cell nuclear transfer (SCNT) to generate cloned piglets. Once the genotype and phenotype of the cloned piglets are confirmed, they supply cells for additional rounds of genetic engineering. FITC-A, fluorescein isothiocyanate. See glossary for additional abbreviations.

Genetically engineered animals for use as human therapeutics, including xenotransplantation, may require multiple genetic modifications throughout their genome to achieve the desired efficacy or therapeutic effect (e.g., immune modulation). A number of groups, including Revivicor, use SCNT to generate genetically engineered early-gestation fetuses (e.g., *Gal* KO fetuses), from which fetal fibroblasts are grown, resetting the Hayflick limit of cell division and thus allowing further steps toward the production of cloned pigs with multiple genetic modification (FIGURE 8). Alternatively, tissues from neonatal or adolescent genetically engineered pigs (ear, kidney, or liver) can be used to derive cells for further rounds of transfection, genetic engineering, and cloning. For example, pigs with homozygous knockout (KO) of the *α1,3GT* gene were further modified with random integration and expression of human complement regulatory genes (*CD55* or *CD46*), anticoagulant genes, or other genes for immune modulation (94).

### 3.4. Multicistronic Vector Technology

Although animals with different single gene edits can be bred to produce offspring that have multiple genetic modifications, each modification segregates independently. Consequently, as the number of modified independent loci increases, the yield of desired offspring decreases significantly. For example, 1 in 4 (25%) will inherit two unlinked modifications, 1 in 16 (6.25%) will inherit three unlinked modifications, and only 1 in 256 progeny (0.4%) would inherit four independently segregating modifications. Multicistronic vector technology solves this issue by allowing for the incorporation and expression of multiple transgenes from a single locus.

Multicistronic vector technology is based on a mechanism used by many eukaryotic viruses to efficiently coexpress multiple gene products from a single open reading frame. Originally identified in the genome of the foot-and-mouth disease virus, specific peptide sequence motifs, consisting of 18–25 amino acids and located between coding sequences, were found to mediate polypeptide cleavage during translation (95). As these amino acid sequences, termed “2A” or “2A-like” sequences, are assembled into the growing polypeptide chain, interaction between the 2A peptide sequence and the ribosome results in the termination of translation and release of the upstream protein. Translation then restarts at the COOH-terminal end of the 2A peptide for synthesis of the next protein. This process solely relies on the peptide sequence, instead of proteinases or mRNA structural elements such as stop codons.

**FIGURE 9.**
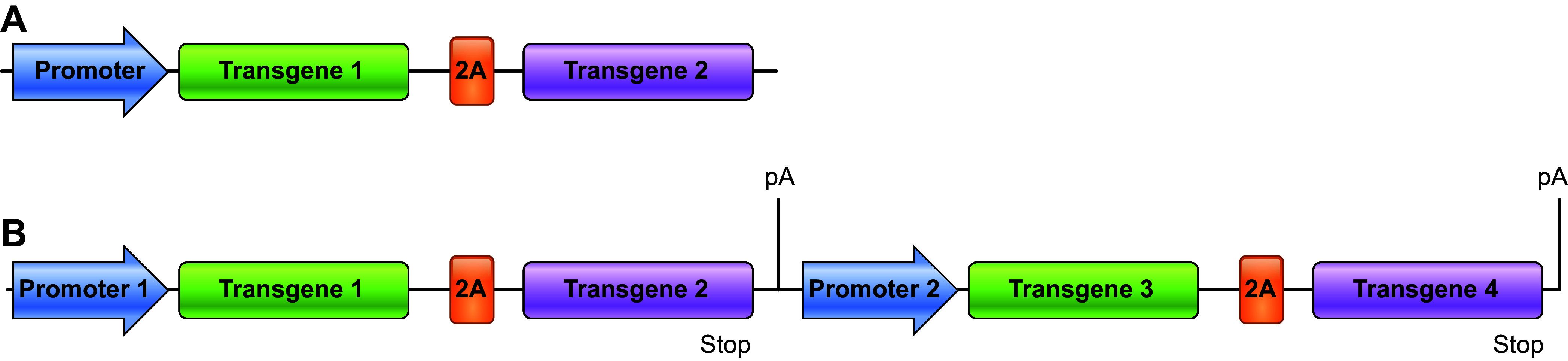
Design of bi- and multicistronic targeting vectors to link transgenes. *A*: bicistronic transgene vector in which 2 transgenes are linked by a 2A sequence to permit expression by a single promoter. *B*: tetracistronic transgene vector composed of 2 bicistrons. The first 2 transgenes are linked by a 2A sequence and expressed by a single promoter. Downstream is a second bicistron containing 2 transgenes linked by a 2A sequence and driven by a single promoter. Each group of 2 transgenes ends with a poly A tail. pA, polyadenylation.

A vector containing two or more transgene coding sequences separated by these “self-cleaving” 2A sequences can be used to generate animals that inherit all transgenes as a single Mendelian locus (FIGURE 9). Because the transgenes are physically linked, they are integrated into the genome together, and thus segregate as one unit in subsequent generations via breeding. As such, the frequency of obtaining animals with the desired genotype (i.e., multiple genetic modifications) is much higher than that from breeding animals with separately generated unlinked modifications (96). Multicistronic vectors containing two sets of transgenes, each set of two controlled by a single promoter, have produced animals with four human transgenes under control of two different promoters (97). As a result, all four transgenes are physically linked in the resulting pigs and thus transmitted together in the resulting progeny.

**FIGURE 10.**
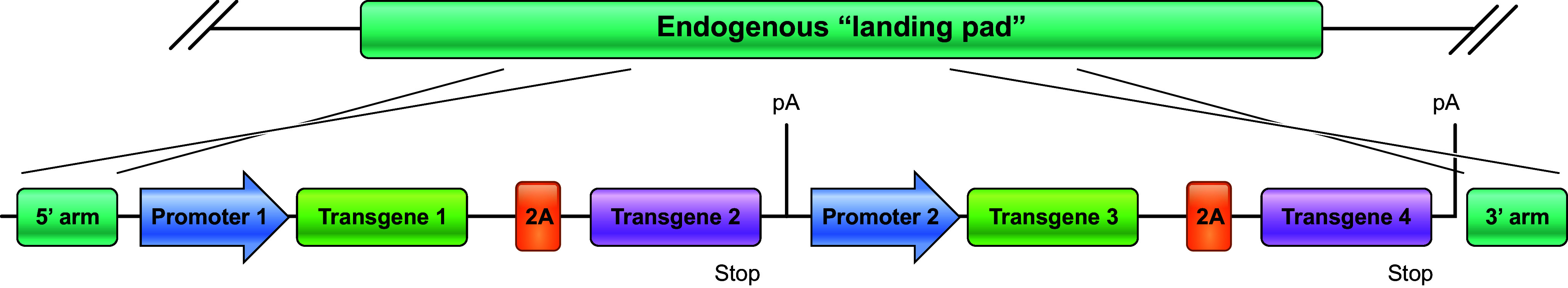
Design of multicistronic targeting vector for targeting to specific “landing pads.” Tetracistronic transgene vector composed of 2 bicistrons. The first 2 transgenes are linked by a 2A sequence and expressed by a single promoter. Downstream is a second bicistron containing 2 transgenes linked by a 2A sequence and driven by a single promoter. Each group of 2 transgenes ends with a poly A tail. This vector is flanked with homology arms to facilitate targeted insertion into landing pads at a specific genomic locus by homology-directed repair (HDR). pA, polyadenylation.

Although the use of multicistronic vectors allows for more efficient generation of multitransgenic animals, the issues associated with random integration of transgenes described in sect. 3.1.1 still apply, including inadvertent inactivation of endogenous genes, aberrant expression of downstream genes, and interference of transgene expression by repressive chromatin. Fortunately, such issues can be avoided by designing multicistronic vectors with homology arms for targeting specific “landing pads” with CRISPR/Cas and homologous recombination. Such landing pads may include sites that are known to be permissive for transgene expression or in specific genes to be knocked out (FIGURE 10). Revivicor has generated pigs with six human multicistronic transgenes, none of which is randomly inserted into the genome as all are integrated via homologous recombination into specific landing pads within the genome (98).

## 4. EVOLUTION OF THE 10 GE PIG: MITIGATION OF MAJOR IMMUNOLOGICAL DIFFERENCES AND MOLECULAR INCOMPATIBILITIES FOR XENOTRANSPLANTATION

Section 3 summarizes the history of each technological advancement required to generate genetically engineered pigs for study in xenotransplantation. This section describes the 25-year iterative approach to determine which combination of porcine genetic modifications will most benefit pig-to-primate xenotransplantation. Although an acceptable “product pig” source will only be realized after testing in human clinical trials, studies in NHP models have provided guidance on which genetic modifications might be advantageous in mitigating immunological and physiological barriers. Over the years, investigators have achieved prolonged pig xenograft survival in NHPs by overcoming formidable xenogeneic immune barriers involving components of both innate and adaptive immune systems.

The first set of xenotransplantation experiments using genetically modified pigs to source organs for NHP recipients were conducted using pigs with randomly integrated single human transgenes. The aim of these studies was to determine whether expression of a human complement regulatory protein in the pig donor increased xenograft survival relative to that seen with wild-type pig organs. The next set of studies examined the impact on xenograft survival of inactivating the *α1,3GT* gene (i.e., deleting the αGal antigen) in the source pig. Finally, studies evaluating the potential synergistic effect on xenograft survival of multiple gene deletions and human transgene insertions in the source pig ultimately led to the evolution of a genetically engineered pig with 10 genetic modifications (10 GE), which includes the inactivation of four endogenous porcine genes and insertion of six human transgenes. These 10 GE donor pigs generated by Revivicor have now been used to provide xenografts for NHP recipients (284), human decedents in preclinical studies (99–101), and two human patients in the first two clinical cases of pig-to-human heart xenotransplantation (102, 103).

### 4.1. Expressing Human Complement Regulatory Proteins in the Pig

As discussed in sect. 3.1.1, transgene insertion via direct injection of DNA fragments into the nuclei of single-celled embryos was the first method established to genetically alter pigs (50). Since endogenous porcine CRPs were shown to be incompatible with primate complement pathway components and insufficient in suppressing NHP complement activation and HAR upon wild-type pig-to-NHP xenotransplantation (17), several groups sought to generate transgenic pigs expressing human-derived CRPs (FIGURE 4).

The first transgenic pigs generated for xenotransplantation were those expressing human CRPs including CD55 (decay accelerating factor), CD46 (membrane cofactor protein), and CD59 (membrane attack complex inhibitor protein) (27, 53, 54, 104–118). CD55, CD46, and CD59 are widely expressed extracellular membrane-associated proteins that act on critical components of the complement cascade pathway to negatively regulate complement activation and thereby prevent damage to healthy cells. The hypothesis was that human CRPs expressed on the surface of porcine cells within the xenograft would sufficiently inhibit recipient (human and NHP) complement pathway components, mitigate formation of the membrane attack complex, and ultimately protect porcine xenograft cells from complement-mediated injury.

### 4.2. Transplantation Studies in NHPs Using Pig Organs That Express Human Complement Regulatory Proteins

Once these human CRP (hCRP) transgenic source pigs were available, multiple groups evaluated the impact of hCRP transgene expression on porcine xenograft survival in pig-to-NHP transplantation. The results were mixed: although organs expressing hCRPs evaded HAR, most failed within 90 days (20-day maximum median survival) (116–119). However, adjunctive therapies modestly improved xenograft survivals from hCRP transgenic donor pigs, and two independent groups achieved isolated maximum graft survivals of 137 (96-day median) and 139 (27-day median) days after heterotopic heart transplantation (grafting a donor non-load-bearing heart into the abdomen of the recipient while the recipient’s heart remains in place) from *hCD55* (105) or *hCD46* (104) transgenic donor pigs.

When *hCD55* transgenic pig hearts were heterotopically transplanted into baboons and a Gal glycoconjugate was administered to neutralize preformed anti-Gal antibodies, Kuwaki and colleagues (105) demonstrated a median graft survival of 27 days (range 4–139 days; *N* = 10). Graft failure occurred in eight animals because of AMR, all of which exhibited hemorrhage, edema, and TMA on histology. Two animals died without graft failure: one from pneumonia and the other because of graft thrombosis and rupture (105). All animals underwent thymic irradiation, and induction immunosuppressive therapy included antithymocyte globulin (ATG) for T-cell depletion and CVF or soluble complement receptor type 1 for complement depletion. Maintenance immunosuppression included mycophenolate mofetil (MMF) to suppress B- and T-cell response and methylprednisone (MP) for inflammation. Heparin was also administered throughout the follow-up period. Additionally, an anti-CD154 MAb was used to block the CD40 costimulation pathway (described in sect. 2.1).

McGregor and colleagues (104) reported a median graft survival of 96 days (range 15–137 days; *N* = 7) of *hCD46* transgenic pig hearts heterotopically transplanted into baboons also using a Gal glycoconjugate. Two xenografts failed because of DXR on posttransplant *days 96* and *137*; histology revealed coagulative necrosis with large areas of ischemic tissue and microvascular thrombosis. The remaining five animals died without graft failure (i.e., grafts were still contracting near the time of death): two because of hemorrhage and one each of renal insufficiency, pulmonary embolism, and a procedural error. All animals underwent splenectomy, and induction immunosuppressive therapy included ATG and anti-CD20 (rituximab) for B-cell depletion. Maintenance immunosuppression included tacrolimus [to inhibit calcineurin (CNI)], sirolimus (to inhibit fibrotic changes and T-cell proliferation), and steroids (to treat inflammation). No postoperative anticoagulants were used.

These studies demonstrated that the expression of human CRPs in the xenografts could protect xenografts from HAR and that adjunctive therapies including the use of Gal glycoconjugates and effective immunosuppression regimens (either CD40 blockade or conventional tacrolimus-based therapies) could extend xenograft survivals after pig-to-NHP heterotopic heart transplantation up to 3 mo. Still, the xenografts ultimately succumbed to DXR with TMA.

### 4.3. Removal of the Major Carbohydrate Antigen (αGal) from the Pig

A major breakthrough in xenotransplantation occurred in the early 2000s when two Nobel Prize-winning technologies, gene targeting and SCNT, merged to produce pigs with an inactivated *α1,3GT* gene (*Gal* KO) and undetectable αGal sugar residues on cells (57, 120–122). As the αGal antigen was considered the major impediment to pig-to-primate xenotransplantation, this milestone was highly anticipated.

Revivicor’s homozygous *Gal* KO (GalSafe^TM^) pig was the first animal with an intentional genetic alteration to receive US Food and Drug Administration (FDA) approval both for human food consumption and as a source for potential therapeutic uses (123). PPL Therapeutics/Revivicor used classical gene targeting technology and cloning via somatic cell nuclear transfer to generate *Gal* KO pigs (FIGURE 5 AND FIGURE 6). A promoter trap targeting vector was constructed from a fragment of porcine DNA that included the *neoR* gene inserted into exon 9 (exon 9 was chosen for disruption since it includes most of the coding sequences for the *α1,3GT* gene, including its catalytic domain). Fibroblasts from standard domestic Large White pigs were transfected with the knockout vector to establish cell lines containing the genetic alteration, such that one allele of the *α1,3GT* gene had been functionally inactivated via targeted insertion of the *neoR* gene sequence (57). Heterozygous cell lines were selected as the source of cells for SCNT, which resulted in several litters of heterozygous founder pigs (57).

A homozygous herd of pigs was obtained through typical breeding practices using heterozygous and homozygous *α1,3GT* knockout pigs. PCR and flow cytometry confirmed the biallelic disruption of the *α1,3GT* gene and absence of the αGal epitopes in the homozygous animals, respectively. Genotyping and phenotyping lineage progenitors to the F_14_ generation confirmed that the genetic modification was stably transmitted to progeny through normal breeding and conformed to Mendelian inheritance.

### 4.4. Transplantation Studies in NHPs Using α1,3GT Knockout Pig Organs

The earliest studies using *Gal* KO pig donors for xenotransplantation into NHP recipients were conducted by separate groups using different lines of pigs (124–128). These groups each demonstrated improved heart and kidney xenograft survivals relative to earlier experiments with wild-type pig organs, including avoidance of HAR in most cases, and, in select studies, isolated long-term xenograft survivals. However, these studies broadly recapitulated the results of experiments with organs from hCRP transgenic source pigs plus Gal glycoconjugates: all xenografts were rejected within weeks to months, as detailed below.

Kuwaki and colleagues (125) transplanted *Gal* KO pig hearts heterotopically into baboons (*N* = 8) using the same immunosuppressive therapy as used when transplanting with *hCD55* transgenic pig hearts (described in sect. 4.2) (105). HAR was avoided; however, five grafts failed between *day 59* and *day 179* (median 78 days) because of AMR. TMA seen in these *Gal* KO hearts was similar to that seen previously when this group transplanted *hCD55* transgenic hearts into baboons (105). Several baboons had IgG deposition in the xenografts. Three animals died without graft failure: one because of bleeding and two that were euthanized for anemia and ischemic limb (125, 126, 129).

McGregor and colleagues (127) also transplanted *Gal* KO pig hearts heterotopically into baboons (*N* = 6) and achieved a 21-day median survival (range of 0–128 days) with conventional immunosuppressive therapy similar to that used when transplanting *hCD46* transgenic pig hearts (described in sect. 4.2) (104). One xenograft failed because of HAR and four because of DXR, which was accompanied by an increased serum concentration of anti-pig (non-Gal) antibody. Additionally, one animal died from hemorrhage after a vascular rupture on *day 2*. Of note, all recipients in this study had higher levels of preformed non-Gal reactive antibodies in contrast to those transplanted by Kuwaki and colleagues as described above (125), which may have contributed to the differences in xenograft survival time between these two studies.

The results were similarly mixed with kidney xenografts from *Gal* KO donor pigs in NHP transplantation experiments. Yamada and colleagues (128) performed the first life-supporting pig-to-baboon kidney transplants using a *Gal* KO donor pig. Treatment included thymectomy, splenectomy, T-cell depletion, anti-CD154 MAb, and MMF, with or without low-dose steroids. Recipients either received kidneys with vascularized thymus (*N* = 11) or kidneys without thymus (*N* = 3). Although recipients of the kidney-thymus combination demonstrated prolonged survival (described in sect. 7.2) the three recipients of kidney grafts without thymus rejected the xenografts between *day 20* and *day 34*, with outcomes similar to those with human *CD55* (*hCD55*) transgenic donor pigs (130). Histology was indicative of the combined effects of antibody and cellular-mediated rejection.

Chen and colleagues (124) described similar results. Baboons (*N* = 6) transplanted with kidneys from *Gal* KO pig donors failed between 8 and 16 days after transplant. Immunosuppression included ATG, tacrolimus, MMF, MP, and CVF in three animals and only ATG, tacrolimus, and MP in the remaining three animals. Although baboon recipients had low concentrations of preformed non-Gal antibodies, anti-porcine antibodies markedly increased after xenotransplantation in four animals, resulting in severe AMR. Histopathology revealed massive interstitial hemorrhage, infarction, necrosis, thrombosis, and loss of tubules with polymorph infiltration and massive deposition of IgG, IgM, C3, C4d, and platelets. Two animals (1 from each immunosuppression treatment group) died from sepsis and hemorrhage (124).

Elimination of the αGal epitope represented an important step forward in that the genetically engineered pig organs were regularly protected from HAR with either conventional tacrolimus-based immunosuppression therapy or immunosuppression including CD40 blockade. But these initial studies with *Gal* KO pigs demonstrated that preformed and elicited antibodies to non-Gal antigens would present additional barriers to long-term graft survival and that additional genetic modifications to the organ source pig, improvements to the treatment regimen, and/or induction of tolerance would be required to further delay xenograft rejection.

### 4.5. Expressing Human Complement Regulatory Proteins in the α1,3GT Knockout Pig

**FIGURE 11.**
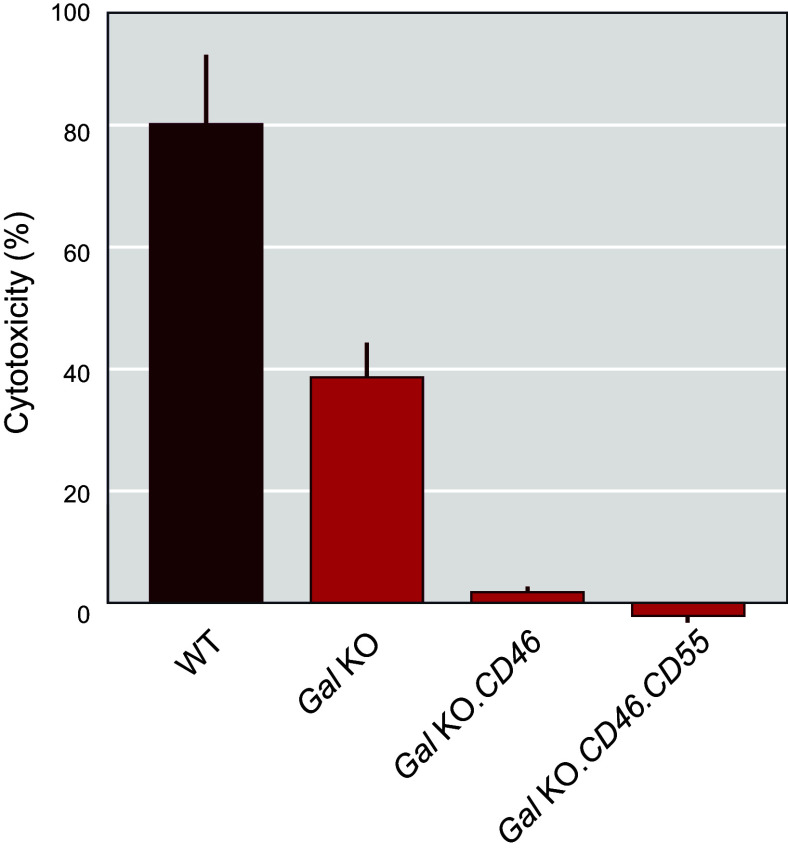
Expression of human complement inhibitors *CD46* and *CD55* in *α1,3GT* (Gal) knockout (KO) cells provides protection from cell lysis. Image-based complement-dependent cytotoxicity (CDC) assay using porcine aortic endothelial cells (pAECs) incubated with 30% pooled human serum (*N* = 3) followed by exposure to 5% rabbit complement for 120 min. Dead cells were stained with Cytotox Red Reagent (IncuCyte), and total cell counts were determined by high-contrast brightfield imaging with a Cytation cell imager (BioTek) to determine % cytotoxicity. Data from 3 replicates are expressed as the % cytotoxicity after 90 min of incubation and compared by ANOVA. WT, wild type. Figure adapted from Ref. 98, with permission from Springer International.

Given the independent benefits of αGal elimination and hCRP transgene expression in donor pigs demonstrated in pig-to-NHP experiments, the logical next step was to develop *Gal* KO donor pigs that also express hCRP transgenes (131). In 2004, Revivicor acquired Australia-based XenoTrans Corporation and imported transgenic pigs containing multicopies of a randomly integrated human complement inhibitor gene *hCD46* (118). These pigs were bred to *Gal* KO pigs to generate offspring with both genetic alterations (i.e., *α1,3GT* knockout plus human *CD46* transgene). The rationale for generating pigs with both modifications was also supported by in vitro cytotoxicity studies using pooled human sera and activated cells from *Gal* KO pigs expressing *hCD55*, *hCD46*, or both transgenes (132). Expression of *hCD55* or *hCD46* demonstrated significant reduction in in vitro cytotoxicity relative to cells with only the *Gal* KO; however, the greatest reduction was seen with cells that express both *hCD55* and *hCD46* (FIGURE 11) (98). Of note, serum from individual NHPs/patients may have low cytotoxicity to cells from pigs with only the *Gal* KO, so that single genetic modification may be sufficient in some cases.

### 4.6. Transplantation Studies in NHPs Using α1,3GT Knockout Pig Organs That Express Human Complement Regulatory Proteins

Combining these genetic modifications proved advantageous in both heart and kidney pig-to-NHP transplantation studies. Although outcomes varied among investigative groups, in general kidney xenografts lasted longer than hearts in these studies, and longer survivals correlated with the use of CD40/CD154 costimulatory blockade as well as selection of recipients with low pretransplant anti-pig antibody levels.

Using hearts from *Gal* KO donor pigs with transgenic expression of *hCD46* (*Gal* KO.*hCD46*), Mohiuddin and colleagues (94) achieved an 8-mo maximum xenograft survival after heterotopic transplantation into baboons (*N* = 15; range of 0–236 days; 100-day median). All baboons received induction with ATG and CVF and maintenance with anti-CD154 MAb, MMF, MP, and anti-CD20 MAb (rituximab). Of note, 12 animals had functional xenografts at the time of death. Two animals in this group experienced DXR. Grafts were histologically normal other than some patchy interstitial fibrosis from animals that lived >100 days. A second group of baboons (*N* = 8) in this report received cardiac xenografts from pigs with the same genetic modifications (*Gal* KO.*hCD46*) and the same immunosuppression regimen, except that this group did not receive rituximab. This group had a maximum survival of ∼30 days (median of 10 days); no grafts exhibited normal function at the time of death. Histology showed signs of DXR including TMA with microvascular thrombosis, interstitial hemorrhage, and ischemic myocyte necrosis. Although both groups had antibody deposition on immunohistochemistry, the group that did not receive rituximab had a marked increase in antibody production during follow-up. Of note, all baboons were bred in a specific pathogen-free (SPF) facility and had low levels of preformed non-Gal antibodies relative to non-SPF baboons (94).

Using separate lines of pigs, McGregor and colleagues (127) transplanted *Gal* KO pig hearts expressing a human *CD55* transgene heterotopically into baboons (*N* = 5) and achieved a 28-day median survival with a range of 15 to 52 days. One animal died from hemorrhage on *day 15*; all remaining grafts failed because of DXR. These results diverged from the outcomes of similar experiments performed by Mohiuddin and colleagues described above. While the hCRP transgene varied between these two studies (Mohiuddin and colleagues used hearts from *Gal* KO pigs expressing *hCD46*, whereas McGregor and colleagues used hearts from *Gal* KO pigs expressing *hCD55*), there were other critical differences. Importantly, McGregor and colleagues did not use CD40/CD154 costimulatory blockade and opted instead for a conventional, CNI-based immunosuppression regimen that is broadly used clinically and that they had used when transplanting *hCD46* transgenic pig hearts (described in sect. 4.2) (104). Moreover, all recipients in this study had higher levels of preformed non-Gal reactive antibodies than those used by Mohiuddin and colleagues (94).

Long-term life-supporting kidney survivals were achieved in a pig-to-NHP xenotransplantation model using a third line of source pigs with a *Gal* KO plus a *hCD55* transgene (133). Of five total rhesus macaques, four selected for having low titers of preformed anti-pig antibodies at transplant underwent T-cell depletion with anti-CD4 and anti-CD8 MAbs, daily MMF and steroids, and either anti-CD154 MAb (*N* = 2) or belatacept (*N* = 2) costimulation blockade. The two animals that received anti-CD154 antibody demonstrated preserved renal function with no evidence of rejection or other pathology on renal biopsies for >126 and 133 days after transplant (1 survived for >10 mo, eventually succumbing to AMR). The two animals who received belatacept rejected their xenografts 14 and 21 days after transplant and exhibited AMR, acute cellular rejection, and TMA. The fifth animal received the regimen including the anti-CD154 MAb but had a high titer of anti-pig antibody and rejected the xenokidney 6 days after transplant, with findings consistent with AMR including interstitial hemorrhage and edema.

These investigators conducted a follow-up study transplanting *Gal* KO.*hCD55* pig kidneys into rhesus macaques with low preformed anti-pig antibodies and immunosuppression of MMF, steroids, and anti-CD154 MAb (134). Six animals also received both anti-CD4 and anti-CD8 MAb, three received anti-CD4 MAb, and three received anti-CD8 MAb. Animals that received anti-CD4 MAb (either alone or in combination with anti-CD8 MAb) had a median survival of 310 days (range 18–499 days), ultimately experiencing late chronic rejection with IgG and C4d deposition. However, those that received only anti-CD8 MAb experienced early rejection with a median survival of 6 days (range 6–13 days), suggesting that CD4 T cells play an important role in xenograft rejection relative to CD8 T cells.

**FIGURE 12.**
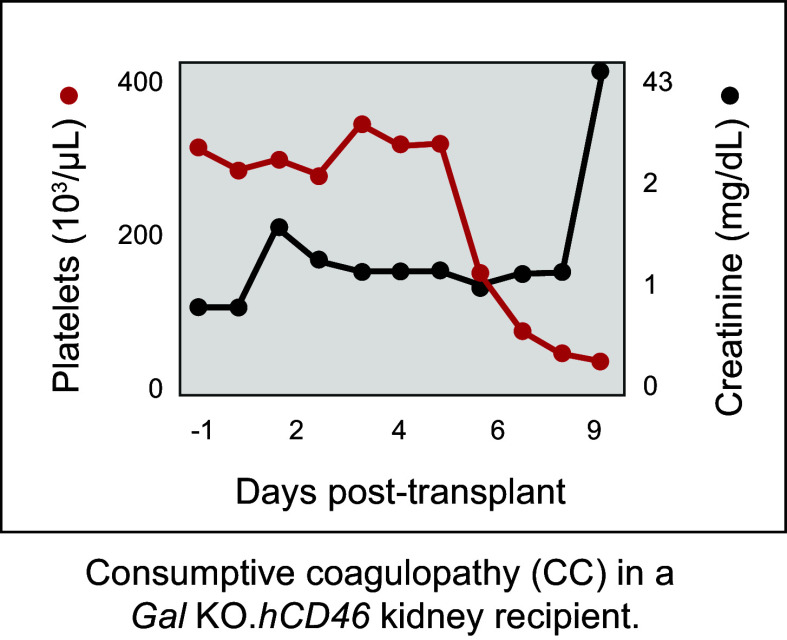
Example of consumptive coagulopathy in a *Gal* KO.*hCD46* kidney recipient. Nonhuman primate (NHP) serum creatinine levels and platelet count after transplantation. Consumptive coagulopathy is indicated by a sudden decrease in platelets followed by an increase in serum creatinine. Image from Revivicor (unpublished) and reprinted with permission from Massachusetts Medical Society.

Taken together, the studies described above using pig-to-NHP xenotransplantation models demonstrated that transgenic expression of a human complement regulatory protein in *Gal* KO pigs can prolong graft survival relative to either modification alone out to median survivals of ∼3 mo for heterotopic heart transplants and 10 mo for kidney transplants, at least in NHPs with low preformed antibody titers that receive immunosuppressive regimens featuring CD40/CD154 costimulatory blockade (94, 133, 134). However, the eventual failure of the xenograft was accompanied by coagulation dysregulation as manifested by TMA that could lead to consumptive coagulopathy in the recipient (FIGURE 12). The next step was to generate pigs that also include human anticoagulant transgenes [i.e., human thrombomodulin (*hTHBD*) and human endothelial protein C receptor (*hEPCR*)].

### 4.7. Expressing Human Coagulation Regulatory Proteins in the α1,3GT Knockout Pig

Thrombomodulin (THBD) is a multidomain, multifunctional glycoprotein expressed primarily on the luminal surface of endothelial cells. Structurally, THBD is composed of extracellular, transmembrane, and intracellular domains. Anticoagulant and anti-inflammatory functions have been ascribed to THBD, both of which are beneficial to xenotransplantation. To maintain hemostasis and prevent clotting, membrane-bound THBD binds circulating thrombin to inhibit its interaction with fibrinogen and other circulating procoagulant proteins. In addition, THBD-bound thrombin has an increased affinity for Protein C and enhances its conversion to Activated Protein C >1,000-fold relative to unbound thrombin. Activated Protein C generation is further enhanced if Protein C is bound to the extracellular protein C receptor (EPCR), another multidomain, multifunctional protein expressed on the luminal endothelial membrane. Activated Protein C exerts its anticoagulant effect mainly through proteolytic deactivation of the amplifying clotting factors Va and VIIIa. Whereas porcine THBD can bind human thrombin, the porcine THBD:human thrombin complex is a very poor activator of human Protein C (135). Pig-to-NHP xenotransplantation data suggest that this incompatibility permits a procoagulant state in the transplanted organs, resulting in TMA and consumptive coagulopathy (27).

**FIGURE 13.**
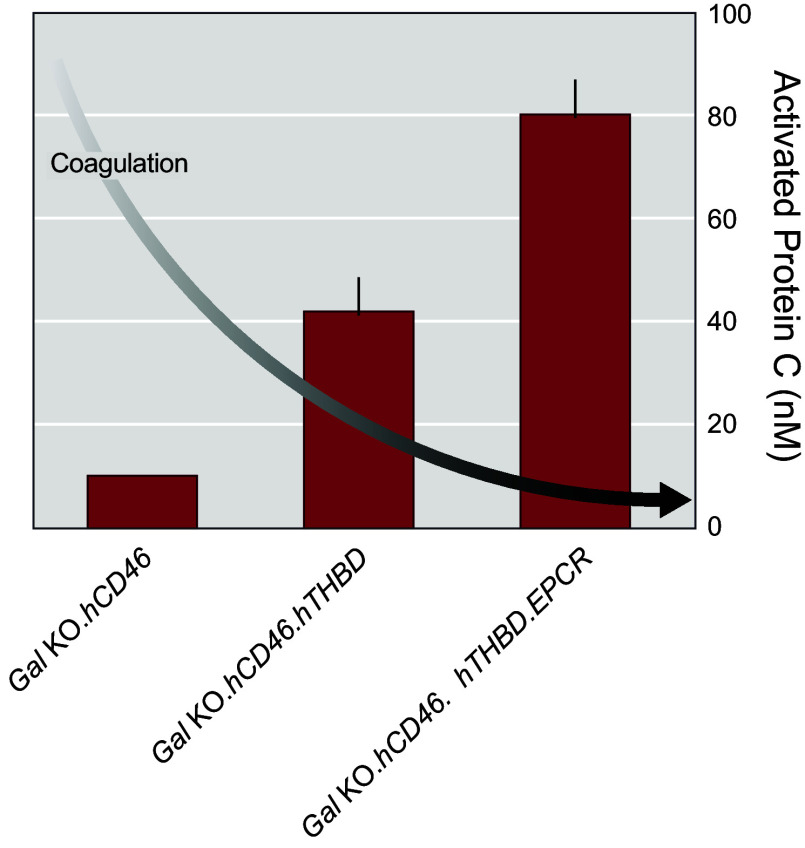
Expression of human anticoagulant transgenes in *Gal* knockout (KO) cells activates protein C to inhibit coagulation. The bioactivity of human thrombomodulin (hTHBD) in porcine aortic endothelial cells, with and without human endothelial protein C receptor (hEPCR), was evaluated by testing its ability to complex with human thrombin and activate human Protein C in vitro. Activated Protein C cleaves a colorimetric substrate that is quantified by absorbance. Figure adapted from Ref. 98, with permission from Springer International.

To overcome this interspecies incompatibility, pigs that express hTHBD were generated (97, 136). Multigene *Gal* KO.*hCD46.hTHBD* pigs were then produced whereby *hTHBD* was expressed from a multicopy, randomly integrated vector under control of the endogenous pig endothelium-specific *THBD* promoter and other regulatory elements to ensure appropriate, physiological expression of *hTHBD* in porcine tissues. Additionally, Revivicor generated a bicistronic vector containing *hTHBD* along with the gene for its cofactor, *hEPCR*, as EPCR facilitates the rate of Protein C activation by the THBD:thrombin complex. The *hTHBD.hEPCR* bicistron was expressed by the same porcine *THBD* promoter to ensure proper expression, including the prevention of overexpression that could cause a bleeding phenotype. The bioactivity of hTHBD in porcine tissues with and without hEPCR was evaluated by testing its ability to complex with human thrombin and activate human Protein C in vitro. Expression of *hTHBD* increased human Protein C activation, which was further enhanced by the addition of *hEPCR* (FIGURE 13) (98). Additionally, pAECs from both multicopy *hTHBD*-expressing pigs and *hTHBD.hEPCR* pigs were shown to activate Protein C and prevent the loss of platelets associated with consumptive coagulopathy in ex vivo lung perfusion studies (137).

### 4.8. Transplantation Studies in NHPs Using α1,3GT Knockout Pig Organs That Express Human Coagulation Regulatory Proteins

The addition of *hTHBD* transgenes to *Gal* KO source pigs expressing human *CD46* (*Gal* KO.*hCD46.hTHBD*) resulted in marked improvements in survival of heart and kidney xenografts after pig-to-NHP transplantation. The results were particularly striking in pig-to-NHP heart transplantation, as Mohiuddin and colleagues (138, 139) achieved a maximum xenograft survival of >2.5 yr after heterotopic cardiac xenotransplantation in NHPs. The immunosuppression regimen used included induction with ATG, rituximab, anti-CD40 Mab, and CVF. Maintenance immunosuppression included MMF, anti-CD40 MAb, and MP to suppress inflammation. All recipient baboons also received continuous heparin infusion to maintain activated clotting time levels at twice the baseline value and aspirin to prevent platelet aggregation.

Before this set of experiments, the only anti-CD154 MAb available to block CD40 costimulation also caused platelet aggregation and further aggravated thromboembolic complications in both nonclinical and clinical studies (140, 141). However, once a primatized antibody against the CD40 molecule was developed (142), Mohiuddin and colleagues (139) tested this antibody in their pig-to-NHP xenotransplantation model. When high-dose anti-CD40 MAb (2.5 times that used for the anti-CD154 MAb) was included in the immunosuppression regimen, five NHP recipients that underwent heterotopic transplantation of *Gal* KO.*hCD46.hTHBD* hearts survived with robust contractility between 159 and 945 days (median 298 days) (138). Some recipients even demonstrated donor-specific unresponsiveness on immunological assays, prompting Mohiuddin and his team to investigate whether this was durable tolerance that might enable long-term survival without immunosuppression. Although xenograft function remained unchanged with small reductions in anti-CD40 MAb doses 1 yr after transplant (*N* = 2), complete cessation of anti-CD40 therapy led to rejection with characteristic features of TMA, vasculitis, intravascular thrombus, myocardial necrosis, epicardial hemorrhage, and a rapid rise of serum anti-pig antibodies (138).

Translation of the success with heterotopic cardiac xenografts to the more clinically relevant orthotopic (life supporting) pig-to-baboon model was not straightforward, and the initial orthotopic transplants failed within a few days (143), despite using the same *Gal* KO.*hCD46.hTHBD* donor pig hearts and a similar immunosuppression regimen (138). Four of five orthotopic cardiac xenografts failed between 1 and 3 days because of severe systolic left heart failure, consistent with the syndrome termed perioperative cardiac xenograft dysfunction (PCXD) (144). The one 30-day survivor succumbed to diastolic left ventricular failure. These five cardiac xenografts had been stored for ∼2 h in static preservation before transplant, suggesting that the addition of a systemic hemodynamic load may exacerbate the consequences of acute ischemia-reperfusion injury during static cold preservation and possibly impair xenograft recovery.

Längin and colleagues were able to mitigate PCXD in the next group of pig-to-baboon xenotransplants by using a nonischemic continuous perfusion (NICP) system (143). This method of NICP was first described by Steen and colleagues (145). When NICP was performed, three of four animals survived 18, 27, and 40 days (1 died on *day 1* because of technical difficulties). However, aberrant cardiac growth and diastolic heart failure occurred (143). By decreasing the baboon blood pressure, reducing cortisone exposure, and administering the mammalian target of rapamycin (mTOR) inhibitor temsirolimus in a third group of animals, they suppressed the aberrant cardiac growth and diastolic heart failure, and two of three animals lived in good health until their scheduled euthanasia at *day 90*. The third animal had occlusion of the thoracic lymph duct and was euthanized at *day 51*. Incorporation of these key steps into the xenotransplantation protocol extended survival of the orthotopic cardiac xenografts >6 mo (*N* = 2) (143).

Mohiuddin and colleagues (146) also extended cardiac xenograft survival in the orthotopic pig-to-baboon model by using a NICP system (XVIVO Heart Preservation System). Xenografts in NHPs (*N* = 4) from source *Gal* KO.*hTHBD.hCD46* pigs survived up to 57 days (median of ∼16 days) with their standard immunosuppression regimen, including CD40 blockade (without temsirolimus or other drugs to control cardiac growth and blood pressure). Histological and immunohistochemistry examination demonstrated endocarditis, monocyte and neutrophil infiltration, fibrin thrombi, an elevated serum non-Gal antibody titer, as well as IgG, IgM, and C4d deposition suggesting AMR.

The benefit of adding human anticoagulant transgenes in *Gal* KO donor pigs was also demonstrated in pig-to-NHP kidney xenotransplantation. However, the relatively few animals in these studies and the use of baboons rather than macaques (which were recipients in the experiments detailed in sect. 4.6) complicate direct comparisons between transplantation with *Gal* KO donor pigs expressing hCRP as well as anticoagulant transgenes and *Gal* KO donor pigs expressing hCRP transgenes alone. In one study, Iwase and colleagues (147) demonstrated survival of a baboon (*N* = 1) out to 136 days using a *Gal* KO pig donor expressing human transgenes *hCD46*, *hCD55*, *hTHBD*, *hEPCR*, and *hCD39*, despite the fact that this baboon had high serum levels of anti-non-Gal IgM antibodies before transplant. The immunosuppression regimen included induction therapy of ATG, rituximab, and CVF, and maintenance included anti-CD40 MAb, rapamycin (sirolimus), and MP. Anti-inflammatory medications included tocilizumab (IL-6R blockade) and etanercept (TNF-α antagonist). Aspirin and low-molecular-weight heparin were also administered. Although death was due to infection and septic shock, histology at necropsy (*day 136*) revealed widespread focal hemorrhage, TMA, and C3 and IgG deposition, indicative of DXR.

### 4.9. Expressing an Anti-Inflammatory Protein (HO1) in the Genetically Engineered Pig

Both allotransplantation and xenotransplantation give rise to inflammation that exacerbates HAR, DXR, and coagulation dysregulation in the transplanted organ (148). Moreover, the presence of an allo- or xenoorgan can generate a sustained, systemic inflammatory state in the recipient, which can endanger both transplanted organ and recipient. Although this can be mitigated to some extent by anti-inflammatory agents, the transgenic expression of anti-inflammatory proteins in the transplanted organ may also be beneficial (131).

Organ transplantation inevitably results in ischemia-reperfusion injury, hemolysis, and heme release, which induces Heme oxygenase-1 (HO1) expression (149). The primary function of HO1 is to catabolize heme to form biliverdin, carbon monoxide, and ferrous ion, which display potent anti-inflammatory, antiapoptotic, and cytoprotective properties. Increased expression of HO1 was found to extend xenograft survival in rodents (150) and to limit ischemia-reperfusion injury-induced tissue damage in heart, kidney, lung, and other organs (151, 152).

To evaluate the potential benefit of expressing human *HO1* (*hHO1*) in pigs for xenotransplant, Petersen and colleagues (153) generated cloned pigs transgenic for an expression vector in which *hHO1* was driven by the SV40 promoter. Human *HO1*-expressing kidneys were then procured and compared with wild-type kidneys in ex vivo perfusion experiments and demonstrated increased survival when perfused with human blood. In addition, *hHO1*-expressing kidneys synthesized fewer molecular markers of vascular damage, had lower vascular resistance, and avoided TMA. Cultured pAECs from *hHO1*-expressing pigs were also largely resistant to TNF-α-mediated apoptosis versus those from wild-type pigs in vitro. These results demonstrated a protective role of transgenic *hHO1* against several hallmarks of HAR and suggested that transgenic *hHO1* could be beneficial in promoting survival of xenotransplanted organs. Human *HO1* was therefore included in Revivicor’s multicistronic vectors to generate pigs expressing *hHO1*.

**FIGURE 14.**
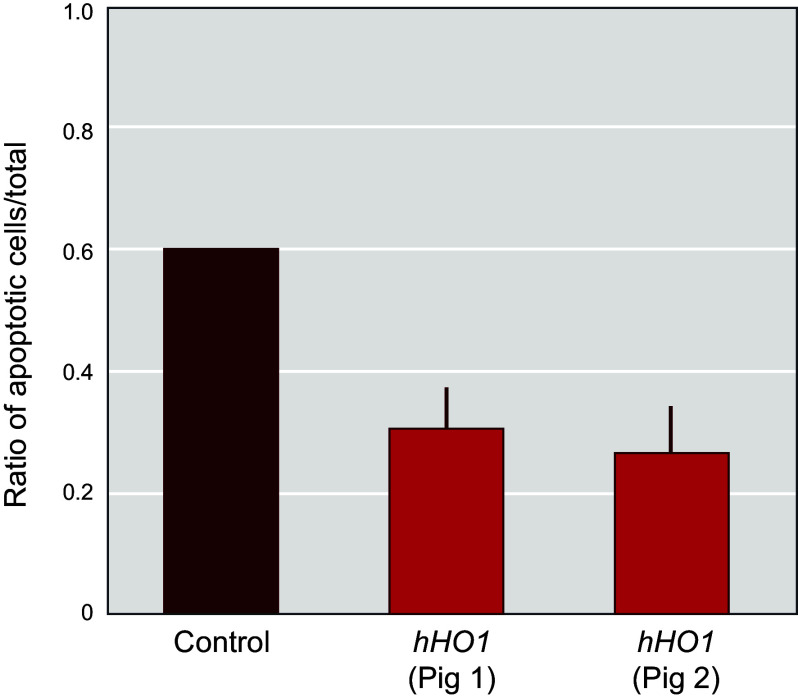
Inhibition of staurosporin-induced apoptosis in cells expressing human heme oxygenase 1 (*hHO1*). Porcine aortic endothelial cells (pAECs) from pigs expressing *hHO1* and pAECs from *Gal* knockout (KO) control pigs that do not express *hHO1* were treated with 1 µM staurosporin for 10 h to induce apoptosis. Apoptosis was assessed with a real-time Caspase 3 assay. Figure adapted from Ref. 98, with permission from Springer International.

The function of hHO1 in pAECs from *hHO1* transgenic pigs has also been evaluated in vitro in the context of additional genetic modifications (98). Cells expressing *hHO1* displayed a significant reduction in apoptotic cells versus cells that do not express *hHO1*, indicating a potential benefit to including the *hHO1* transgene in organ source pigs (FIGURE 14).

### 4.10. Expressing a Macrophage Inhibitory Protein (CD47) in the Genetically Engineered Pig

Macrophages are a key cell type in the innate immune system, where they are important for the identification and elimination of senescent, dead, foreign, and other undesirable cell types (microorganisms, virally infected host cells, etc.) from the body. Normal cells avoid detection from autologous macrophages by a ubiquitously expressed surface protein, CD47. CD47 performs a critical “don’t eat me” function that protects host cells from macrophage attack and destruction. The key protective interaction involves the binding of CD47 to a macrophage ligand, SIRP-α, to block phagocytosis (154).

Whereas CD47 is relatively conserved across mammalian species, SIRP-α is not, leading to CD47-SIRP-α incompatibilities and poor binding affinities between certain species, notably pig and human. In pig-to-primate xenotransplantation, pig cells are susceptible to host macrophage phagocytosis due to the inability of porcine CD47 to bind and activate primate SIRP-α (155). Fortunately, this incompatibility may be overcome by transgenic expression of human *CD47* (*hCD47*) on porcine cells to protect them from attack by primate macrophages (156). The *hCD47* transgene was therefore included in Revivicor’s multicistronic vectors to generate pigs expressing hCD47.

**FIGURE 15.**
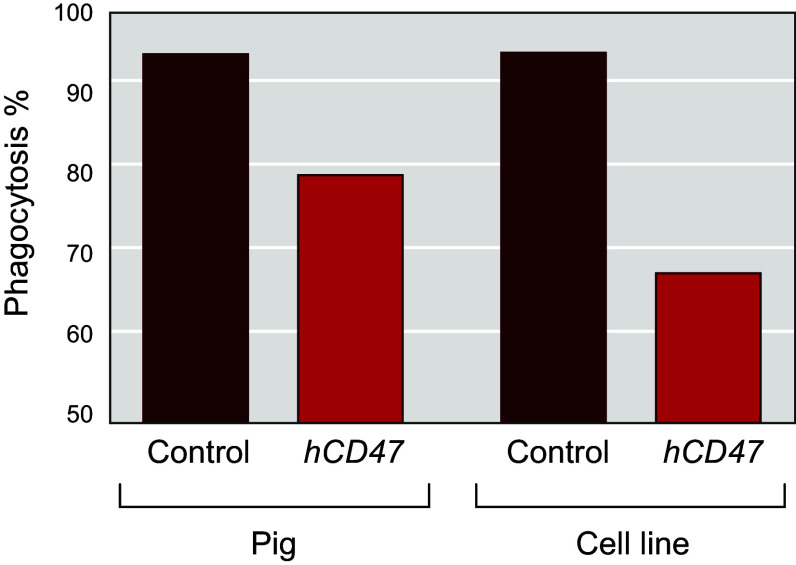
Inhibition of phagocytosis of porcine cells expressing human *CD47* (*hCD47*). Porcine aortic endothelial cells (pAECs) transfected with a *hCD47* expression vector, pAECs from pigs expressing *hCD47*, and pAECs from control pigs that do not express *hCD47* were transfected with a constitutive green fluorescent protein marker. Transfected cells were then cocultured with human macrophages tagged with red and blue fluorescent antibodies to major histocompatibility complex (MHC) class II and CD14, respectively. After 4 h, cells displaying all 3 fluorescent markers were counted as having undergone phagocytosis. Image from Revivicor (unpublished) and reprinted with permission from Massachusetts Medical Society.

The ability of hCD47 to protect pig cells against human macrophage attack was evaluated in vitro with pAECs transfected with a *hCD47* expression vector as well as pAECs obtained from *hCD47* transgenic pigs (FIGURE 15) (131). Cells expressing *hCD47* were less susceptible to human macrophage phagocytosis versus non-*hCD47*-expressing cells.

To date, results of pig-to-NHP transplantation using *hCD47* transgenic pigs have been limited but promising. Pig-to-NHP hematopoietic stem cell (HSC) transplantation using *hCD47* transgenic pigs demonstrated prolonged survival of porcine xenogeneic HSCs in the recipient peripheral blood relative to HSCs that do not express *hCD47*, indicating reduced consumption by recipient immune system; importantly, this prolonged HSC chimerism translated to prolonged survival of cotransplanted skin xenografts (157) and lung xenografts (158). Interestingly, the impact of *hCD47* transgene expression on kidney xenograft survival may depend on where *hCD47* is expressed in the graft. Yamada and colleagues (159) demonstrated that podocyte expression of *hCD47* was associated with prevention of podocyte injury and proteinuria; conversely, they showed that high *hCD47* expression in renal tubular cells may lead to inflammatory changes through activation of the CD47/thrombospondin-1 pathway.

### 4.11. Removal of Additional Major Carbohydrate Antigens (Neu5Gc and SDa) from the Genetically Engineered Pig

The inclusion of human transgenes to inhibit complement and mitigate coagulation dysfunction included in the genome of the organ source *Gal* KO pig, plus more sophisticated immunosuppression therapies, significantly increased xenograft longevity in pig-to-NHP transplant models. However, the eventual development of DXR along with anti-pig antibodies seen by immunohistochemistry analysis of rejected grafts prompted the identification and deletion of additional porcine major carbohydrate antigens.

Two additional porcine major carbohydrate xenoantigens, n-glycolylneuraminic acid (Neu5Gc) (160) and sialyl-dimeric antigen (SDa) (161), were identified that may also contribute to porcine organ rejection, at least in humans. Like αGal, these antigens are terminal residues on sialylated glycans. The synthesis of Neu5Gc is catalyzed by cytidine monophosphate-N-acetylneuraminic acid hydroxylase (CMAH), encoded by the *CMAH* gene. Neu5Gc is present in most mammals including all Old World primates, except for humans, who do not express *CMAH* because of an inactivating mutation that occurred just after the last common ancestor of humans and other great apes (162). Interestingly, New World primates do not express *CMAH* either, because of an independent loss-of-function mutation that occurred after the divergence of Old and New World primates. Similar to αGal, chronic dietary exposure stimulates production of anti-Neu5Gc antibodies in humans, which are expected to contribute to xenograft rejection in humans (163). As such, deletion of the *CMAH* gene in pigs should further reduce AMR upon porcine xenograft transplantation in humans. In contrast, since Old World primates do express *CMAH* (and do not have anti-Neu5Gc preformed antibodies), the impact of deleting the *CMAH* gene on porcine xenograft rejection in humans cannot be determined in Old World primate models, including baboons and macaques.

SDa synthesis is catalyzed by β-1,4-N-acetyl-galactosaminyltransferase 2 encoded by the *β4GalNT2* gene (161). Binding studies have confirmed the presence of preformed SDa antibodies in human sera and have also confirmed the presence of SDa antigen in pig vascular endothelium. In addition to the preformed anti-SDa antibodies, induced anti-SDa antibodies were detected in baboons after porcine organ transplantation. Anti-SDa antibodies have also been induced in humans by antitumor vaccines known to contain SDa. It is likely that anti-SDa antibodies contribute to both HAR and delayed antibody-mediated rejection in baboons as well as humans (161).

To eliminate expression of these antigens in pigs, Revivicor knocked out the *CMAH* gene in their *Gal* KO line, first using TALENS and soon thereafter using CRISPR/Cas9 technology to generate *Gal* KO.*CMAH* double-KO pigs. CRISPR/Cas9 was then used to knock out the *β4GalNT2* gene in cultured *Gal* KO.*CMAH* KO fibroblasts, which underwent SCNT to generate pigs with disruptions in all three genes, or triple-knockout pigs (*Gal* KO.*CMAH* KO.*β4GalNT2* KO) (Revivicor, unpublished observations).

**FIGURE 16.**
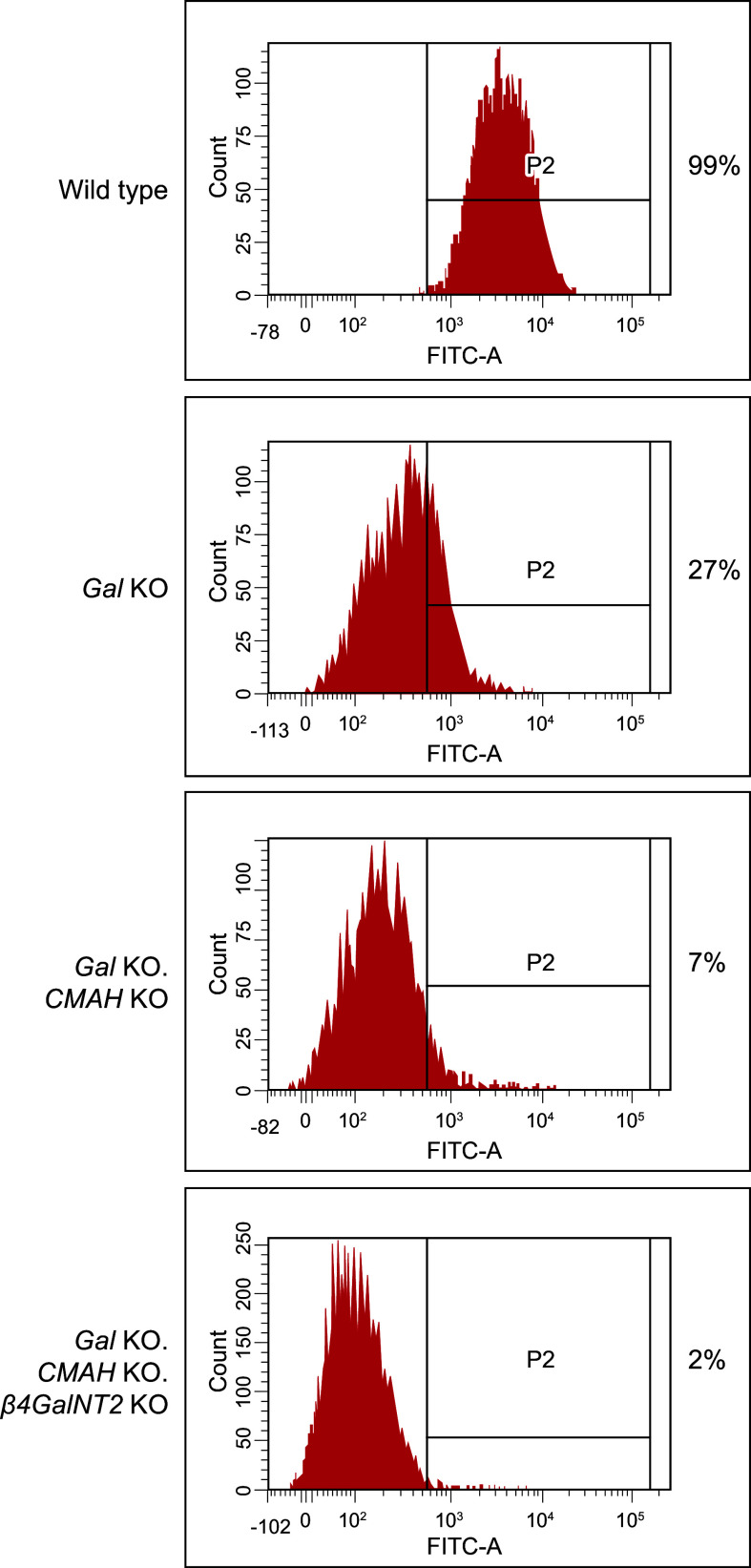
Reduced human serum antibody binding to porcine vascular endothelial cells with disruptions in genes for synthesis of additional major carbohydrate antigens. Porcine vascular endothelial cells were incubated with sera from human donors (*N* = 3), probed with anti-IgG secondary antibody, and counted by flow cytometry. Results are expressed as % immunoglobulin bound relative to wild type. FITC-A, fluorescein isothiocyanate; Ig, immunoglobulin. See glossary for additional abbreviations. Image from Revivicor (unpublished) and reprinted with permission from Massachusetts Medical Society.

The effect of each gene knockout on human serum antibody binding and complement-mediated cell lysis was assessed with porcine vascular endothelial cells from wild-type pigs versus pigs with single, double, and triple gene knockouts. The IgG binding was reduced by 72%, 92%, and 97% with *Gal* KO, *Gal* KO.*CMAH* KO, and *Gal* KO.*CMAH* KO.*β4GalNT2* KO cells, respectively, evaluated by flow cytometry (FIGURE 16) (Revivicor, unpublished observations). Similar results were obtained with different porcine cell types (98, 131). Thus, knockout of these three major xenoantigens eliminated the majority of preformed human serum antibody binding to porcine cells. Indeed, screening of 820 patients on the renal transplant waitlist demonstrated that many have a negative crossmatch to triple-KO cells (164).

**FIGURE 17.**
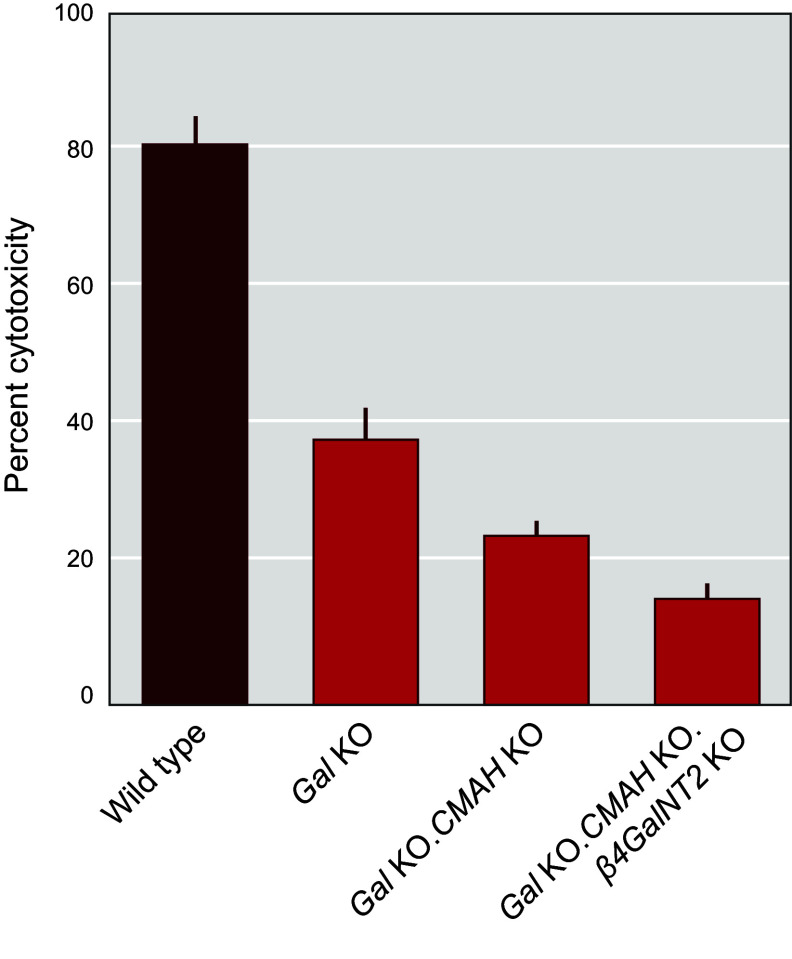
Reduced cytotoxicity with additional carbohydrate deletions. Image-based complement-dependent cytotoxicity (CDC) assay using pAECs incubated with pooled human serum (*N* = 3) followed by exposure to rabbit complement. Dead cells were stained, and total cell counts were determined by high-contrast brightfield imaging. See glossary for abbreviations. Figure adapted from Ref. 98, with permission from Springer International.

The functional cytoprotective effect of these knockouts was assessed by in vitro complement-dependent cytotoxicity (CDC) assays. As shown in FIGURE 17, protection from CDC increased with each additional knockout. A recent study demonstrated that the removal of αGal and/or Neu5Gc from the pig results in altered levels and distribution of other diverse glycans (165). Regardless, antibody binding and CDC assays clearly demonstrate that removing these three porcine carbohydrate antigens significantly decreases the overall immunogenicity of pig cells in human sera.

### 4.12. Removal of the Growth Hormone Receptor from the Genetically Engineered Pig

**FIGURE 18.**
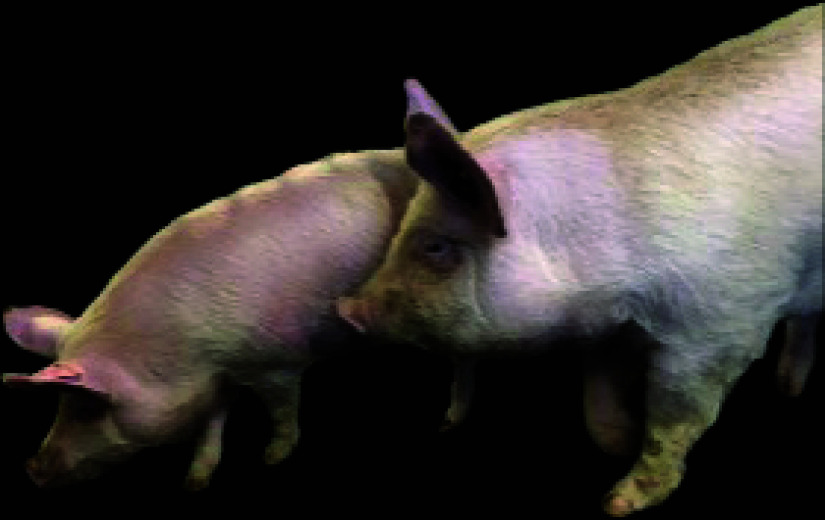
Growth hormone receptor gene knockout results in reduced growth of the pig. *GHr* knockout (KO) pig (*left*) and wild-type pig (*right*). Image from Revivicor.

In 2019–2020, Revivicor efforts were initiated to knock out the growth hormone receptor gene (*GHr*) because of potential concerns that the pig organs from the “Large White” pig lineage might grow too large in human recipients after transplant. CRISPR/Cas9 was used to knock out the *GHr* gene, which proved to be quite efficient, and both alleles were inactivated in a single transfection. The GHr KO not only reduced the size of the pigs by 40% (they do not grow beyond 150 kg) (FIGURE 18) but also resulted in 80–90% reduced expression of endogenous insulin-like growth factor 1 (IGF-1) (146, 166, 167). Although this reduction in IGF-1 causes transient hypoglycemia in young preweaning-age GHr KO pigs, it is not a deleterious phenotype.

### 4.13. Transplantation Studies in NHPs Using Pig Organs with Multiple Gene Knockouts and Multiple Human Transgenes

Unlike the results seen with human serum, which shows decreased antibody binding and CDC to triple- versus double-KO pAECs (FIGURE 16 AND FIGURE 17), sera from baboons and macaques often demonstrate increased antibody binding and CDC to triple-KO (*Gal* KO.*CMAH* KO.*β4GalNT2* KO) versus double-KO (*Gal* KO.*β4GalNT2* KO) pig cells (146, 168, 169). Likewise, results from several NHP transplantation studies using triple-KO pig xenografts indicate that the deletion of the pig *CMAH* gene increases the immunogenicity of the xenograft in baboons and may contribute to rejection (146, 168, 170).

Yamamoto and colleagues (168) assessed xenograft survival in baboons (*N* = 3) that received pig kidneys with deletions of all three major carbohydrate antigens plus human transgenes (*Gal* KO.*Β4GalNT2* KO.*CMAH* KO.*hTHBD.hEPCR.hCD46.hCD55.hCD47.hHO1*) versus baboons (*N* = 5) that received pig kidneys with only the *Gal* KO plus combinations of two to five human transgenes [*hTHBD, hEPCR, hCD46, hCD55, hCD47, hCD39, hHO1*, and/or human von Willebrand factor (*hvWF*)]. Immunosuppression was the same in both groups and included induction with ATG and rituximab and maintenance with anti-CD40 MAb, rapamycin, and low-dose corticosteroids. The baboons that received the triple-KO kidneys had higher anti-pig IgG, lower IgM, and similar CDC at prescreening relative to those baboons that received the *Gal* KO kidneys. Of the three baboons that received triple-KO kidneys, the longest survivor was euthanized on *day 61* because of AMR. The other two were euthanized within 1 wk: one with HAR and the other with gastric dilation. In contrast, four of five baboons that received pig kidneys with only the *α1,3GT* knockout plus transgenes survived between 90 and 260 days (186-day median). Only one baboon had AMR after two doses of anti-CD40 MAb were withheld because of neutropenia. Three required euthanasia for infections with no obvious clinical or histopathological signs of rejection, and one was euthanized on *day 4* because of gastric dilation.

Mohiuddin and colleagues (138, 146) assessed survival of triple-KO pig hearts in an orthotopic pig-to-baboon model using their standard immunosuppression regimen. Graft failure occurred within 1 wk in all three baboons that received triple-KO xenografts [3 GE: *Gal* KO.*β4GalNT2* KO.*CMAH* KO (*N* = 2) or 5 GE: *Gal* KO.*β4GalNT2* KO.*CMAH* KO.*hCD46.hCD55* (*N* = 1)]. On gross postmortem examination, notable intracardiac thrombi were seen with propagation into the aorta, pulmonary arteries, and coronary sinus of some of these xenografts. Histological examination revealed intracardiac organizing thrombus and intravascular fibrin thrombi with regions of myocardial ischemia. However, when baboons (*N* = 2) received triple-KO pig hearts plus six human transgenes that included transgenes for human thromboregulatory proteins (9 GE: *Gal* KO.*β4GalNT2* KO.*CMAH+/−.hTHBD.hEPCR.hCD46.hCD55.hCD47.hHO1*), both baboons still experienced AMR, but survival was increased to 84 and 95 days. One had interstitial edema and hemorrhage, microvascular thrombosis, fibrosis, cellular infiltration, and endotheliosis, and the other had mild interstitial inflammation, chronic xenograft vasculopathy, and a large acute septal infarct. In contrast, the two baboons that received xenografts with deletions of only two of the three major carbohydrate antigens plus four human transgenes for both complement and coagulation regulation (7 GE: *Gal* KO.*β4GalNT2* KO.*GHr* KO.*hTHBD.hEPCR.hCD46.hCD47*) demonstrated markedly prolonged xenograft survival relative to the 3 GE, 5 GE, and 9 GE xenografts described above. One xenograft functioned for 264 days, which is the longest reported life-supporting xenoheart survival to date. The second had to be euthanized because of reduced food intake and weight loss from gingivitis but had excellent cardiac xenograft function per transthoracic echocardiography (TTE) up until the time of euthanasia on *day 182*.

Although the *CMAH* KO-related neoantigen exposure is undoubtedly limiting in pig-to-baboon/macaque xenotransplantation models, recent studies have demonstrated extended xenograft survival times after transplantation of kidneys containing knockouts of all three major carbohydrate antigens plus human transgenes into macaque recipients (171, 172). In a study conducted using source pigs generated by eGenesis, a group of six cynomolgus macaques transplanted with triple-KO pig kidneys and five human transgenes [*hCD46, hCD55, hCD59, hCD47*, and human leukocyte antigen E (*HLA-E*)] survived between 15 and 316 days (median of 103 days) (172). Two lost graft function within 3 wk: one because of vascular thrombosis and the other because of hydronephrosis. Four had graft failure between 71 and 316 days after transplant due to AMR and TMA after immunosuppression was reduced because of infections. Induction immunosuppression included anti-CD20 MAb and ATG. Maintenance immunosuppression included anti-CD154 MAb, MMF, rapamycin or tacrolimus, and MP.

In a second study conducted by the same group, macaques were transplanted with triple-KO pig kidneys (*Gal* KO, *β4GalNT2* KO, and *CMAH* KO) plus seven human transgenes [*hTHBD, hEPCR, hCD46, hCD55, hCD47, hHO1*, and tumor necrosis factor alpha-induced protein 3 (*TNFAIP3*)] with and without inactivation of porcine endogenous retrovirus (PERV) elements (171). Although long-term survival >6 mo was achieved in 7/15 animals (>2 yr in 1 case and >1 yr for 2 others that are still ongoing), 6/15 animals survived <26 days because of renal failure and/or AMR and TMA.

In summary, studies (146, 168, 169) demonstrated that double-KO (*Gal* KO.*β4GalNT2* KO) xenografts survived longer than triple-KO (*Gal* KO.*β4GalNT2* KO.*CMAH* KO) and *Gal* KO xenografts in baboon and macaque recipients. These results, coupled with the in vitro results that demonstrate decreased NHP antibody binding and CDC with cells from double- versus triple-KO pigs, are consistent with the hypothesis that removal of Neu5Gc exposes a neoantigen in the xenograft, which may contribute to AMR in these NHPs (131). Since humans do not produce Neu5Gc, this neoantigen would be naturally exposed. Consequently, triple-KO pig xenografts should be less immunogenic in humans versus those with only a double KO, even if the opposite is true in baboons.

These studies also demonstrated that adequate expression of human thromboregulatory proteins and CRPs in the triple-KO porcine xenograft may mitigate the *CMAH* KO-related AMR in Old World monkeys and increase survival out to >2 yr in a subset of recipients (146, 171, 172). Although no Old World monkeys have a negative crossmatch, choosing recipients with low anti-pig donor antibodies will likely further extend survival of triple-KO xenografts in this NHP model. Indeed, in an ongoing xenotransplantation study in NHPs receiving kidneys from Revivicor pigs with 10 genetic modifications (*Gal* KO.*CMAH* KO.*β4GalNT2* KO.*hCD46.hCD55.GHr KO.hTHBD.hEPCR.hCD47.hHO1*), Eisenson and colleagues have achieved extended longevity (>6 mo) in a cohort of baboon recipients (284). This case series demonstrates for the first time consistent and consecutive long-term survival in an NHP xenotransplantation model after porcine kidney procurement at a remote DPF facility and 3–5 h of hypothermic machine perfusion. Perhaps most importantly, they have achieved long-term xenograft survival in genetically modified pig-to-baboon kidney transplantation using conventional, clinically available immunosuppression. This achievement is likely due to reliable transgene expression in the Revivicor 10 GE source pig as well as a recipient screening methodology developed in the Yamada laboratory (285) to select recipients with low levels of preformed antibodies to the specific donor pig.

### 4.14. The 10 GE Pig

**FIGURE 19.**
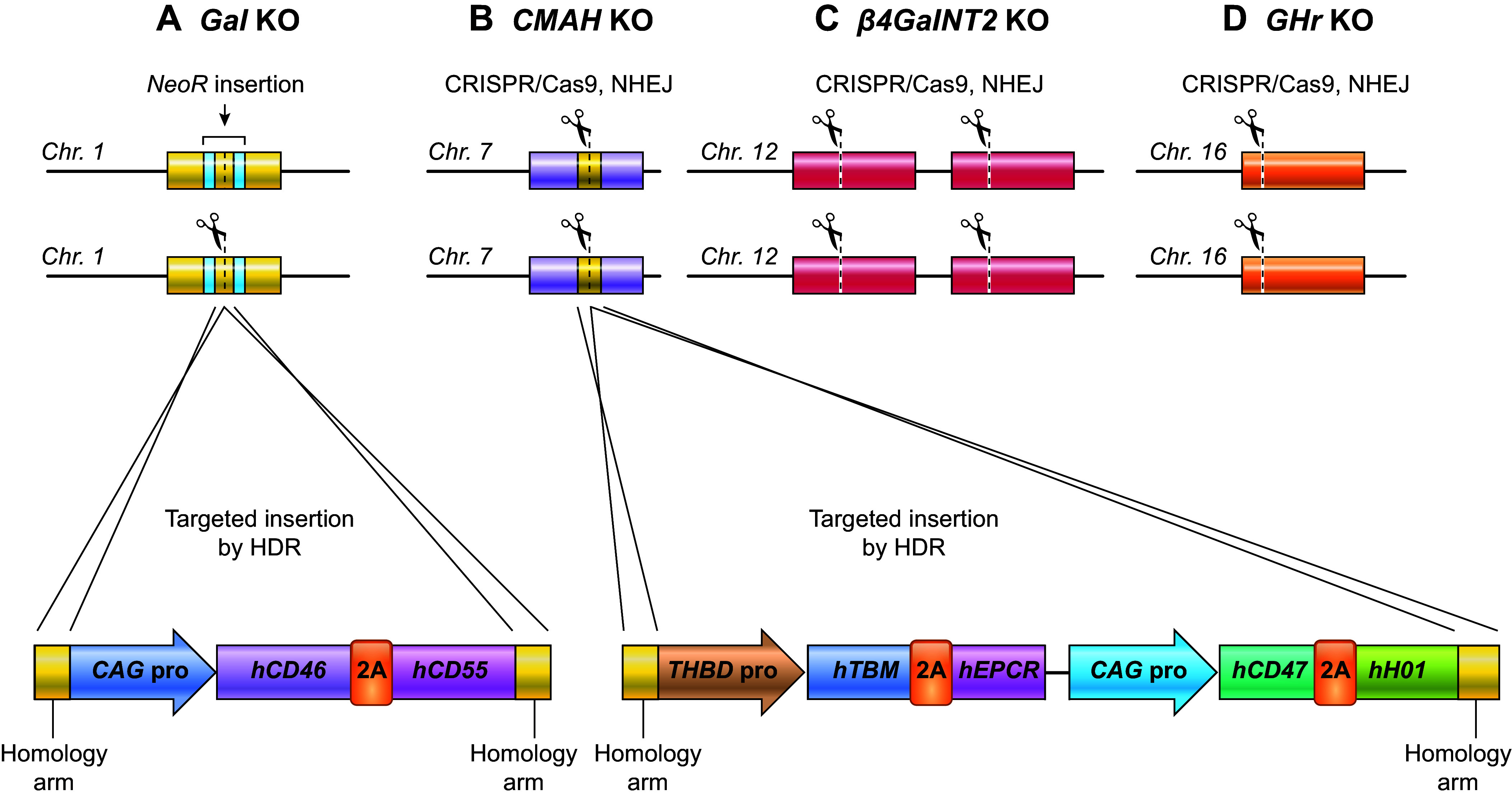
Targeting vector designs used to generate the 10 GE pig. *A*: bicistronic transgene vector in which human (h)*CD46* and *hCD55* are linked by a 2A sequence to permit expression by a single *CAG* promoter. This vector is flanked with homology arms to facilitate targeted insertion by homology-directed repair (HDR) into a landing pad directed to the *α1,3GT* locus. The second allele of the *α1,3GT* gene was inactivated by a *neoR* insertion via homologous recombination. *B*: tetracistronic transgene vector composed of 2 bicistrons. In the first, human thrombomodulin (*hTHBD*) and human endothelial protein C receptor (*hEPCR*) are linked by a 2A sequence and expressed by a single porcine *THBD* promoter. Linked downstream to this is a second bicistron containing *hCD47* and *hHO1*, linked by a 2A sequence and driven by a single *CAG* promoter. This vector is flanked with homology arms to facilitate targeted insertion into landing pads on the cytidine monophosphate-N-acetylneuraminic acid hydroxylase (*CMAH*) locus by HDR. The second allele of the *CMAH* gene was inactivated by CRISPR/Cas9-mediated NHEJ. *C*: inactivation of both alleles of the *β4GalNT2* gene by CRISPR/Cas9-mediated nonhomologous end joining (NHEJ). *D*: inactivation of both alleles of the *GHr* gene by CRISPR/Cas9-mediated NHEJ. Chr, chromosome; *HO1*, heme oxygenase-1; *NeoR*, neomycin resistance. See glossary for additional abbreviations.

After two decades of experimentation in transplanting organs from source pigs containing one or more gene knockouts and one or more human transgene insertions into NHPs, Revivicor deemed the pig with 10 genetic modifications (i.e., the 10 GE pig) the “product pig” to be developed as a biological product under FDA regulations; once approved by the FDA, it would be available for human use. This 10 GE pig has four porcine genes inactivated (*α1,3GT*, *CMAH*, *β4GalNT2*, and *GHr*) and contains a set of six human transgenes, including two human complement inhibitor genes (*hCD46* and *hCD55*) to address remaining pig antigens, two human anticoagulant genes (*hTHBD* and *hEPCR*) to address coagulation dysfunction, *hCD47* (T cell, macrophage inhibition), and *hHO1* (anti-inflammatory). To facilitate the generation of offspring with the desired genotype and to ensure consistent transgene expression, a two-gene multicistronic vector (including human transgenes *hCD46* and *hCD55*) was integrated at the *α1,3GT* locus (or *α1,3GT* landing pad) and a four-gene multicistronic vector (including human transgenes *hTHBD, hEPCR, hCD47*, and *hHO1*) was integrated at the *CMAH* landing pad. As such, all six human transgenes were inserted into only two loci, which also served to knock out one allele of the *α1,3GT* and *CMAH* genes. The second alleles of the *α1,3GT* and *CMAH* genes were inactivated by a *neoR* insertion via homologous recombination and CRISPR/Cas9-mediated NHEJ, respectively. Both alleles of the *β4GalNT2* and *GHr* genes were inactivated by CRISPR/Cas9-mediated NHEJ (FIGURE 19; TABLE 1) (98, 146).

**Table 1.** Summary of targeted gene editing in the 10 GE pig

Gene	Description
*Gal* KO	Inactivation of the porcine *α1,3GT* gene responsible for the synthesis of the antigen αGal
*β4GalNT2* KO	Inactivation of the porcine *β4GalNT2* gene responsible for the synthesis of the antigen SDa
*CMAH* KO	Inactivation of the porcine *CMAH* gene responsible for synthesis of the antigen Neu5Gc
*GHr* KO	Inactivation of the porcine *GHr* gene encoding the growth hormone receptor that regulates growth of the animal
*hCD46-hCD55* insertion	Insertion of the human *CD46* transgene encoding the complement pathway inhibitor protein CD46 (membrane cofactor protein)
	Insertion of the human *CD55* transgene encoding the complement pathway inhibitor protein CD55 (decay accelerating factor)
*THBD-EPCR-CD47-HO1* insertion	Insertion of the human *THBD* transgene encoding thrombomodulin, an inhibitor of coagulation, thrombosis, and platelet aggregation in vascular and microvascular endothelium
	Insertion of the human *EPCR* transgene encoding the EPC receptor, which has anticoagulant activity
	Insertion of the human *CD47* transgene encoding the CD47 protein, which inhibits macrophage responses
	Insertion of the human *HO1* transgene encoding heme oxygenase 1, which has anti-inflammatory properties.

See glossary for abbreviations.

**FIGURE 20.**
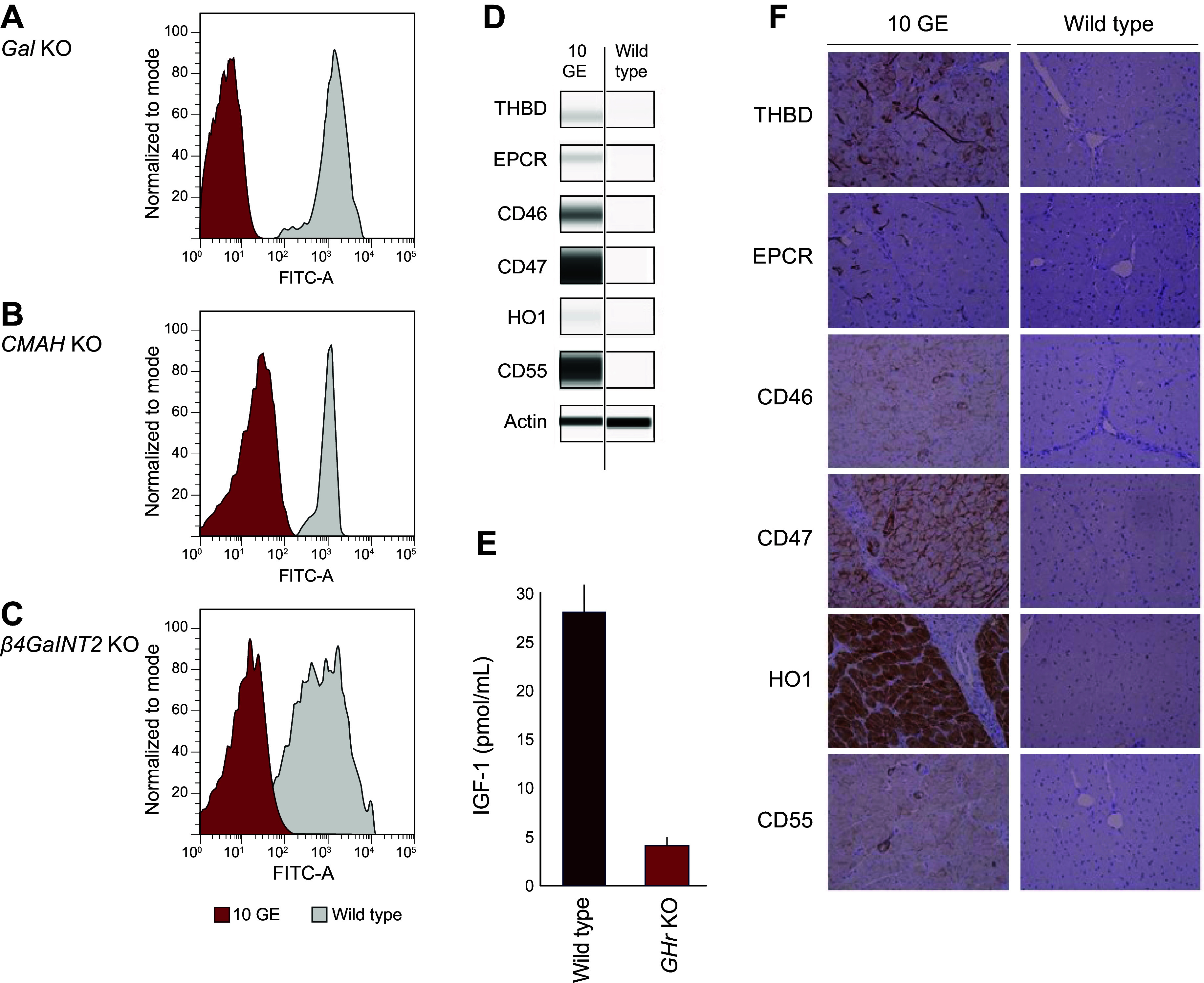
Representative phenotypic identity of 10 GE pigs. *A–C*: flow cytometry confirming the absence of αGal (*A*), Sda (*B*), and Neu5Gc (*C*). *D*: Western blot of human transgene expression in pig tissue. *E*: serum IGF-1 levels in *GHr* KO donors vs. wild-type pigs. *F*: immunohistochemical detection of human transgene products in pig tissue. FITC-A, fluorescein isothiocyanate. See glossary for additional abbreviations. Figure adapted from Ref. 146, with permission from *Xenotransplantation*.

Before transplant, the genotype and phenotype of each donor pig are confirmed. PCR, NextGen DNA sequencing, and Southern blot methodologies with tail biopsy DNA from each product pig are used to confirm the presence of all intended genetic modifications and to rule out any unintended transformation events such as insertion of additional/random copies of the human transgenes or the vector backbone or off-target CRISPR cutting. Flow cytometry is used to confirm the absence of the αGal, SDa, and Neu5Gc epitopes, and Western blot and immunohistochemistry confirm the expression of human gene products in tissue specimens. Pig size and the determination of the IGF-1 concentration confirm the absence of GHr (FIGURE 20).

In addition to Revivicor, other companies in both the private and public sectors have generated genetically engineered pigs with multiple gene inactivations and/or transgene insertions whose organs may be used for xenotransplantation. The National Swine Resource and Research Center (University of Missouri-Columbia, Columbia, MO) has several strains of genetically modified pigs that can be purchased for use as models in biomedical research (https://nsrrc.missouri.edu). eGenesis (Cambridge, MA; https://egenesisbio.com), Makana Therapeutics (Miami, FL; https://makanatherapeutics.com), and XTransplant (https://xtransplant.com) are currently testing organs from their genetically modified pigs in the pig-to-NHP xenotransplantation model with the intent of developing these xenoorgans for human xenotransplantation.

## 5. ANATOMY AND PHYSIOLOGY OF THE PORCINE HEART AND KIDNEY

### 5.1. Heart

The similarity between the wild-type pig and human hearts regarding the major blood vessels, coronary arteries, heart valves, and the cardiac conduction system is remarkable. However, subtle differences exist that impact xenotransplantation techniques and may impact long-term follow-up procedures after xenotransplantation (173, 174). The main anatomical differences between pig and human hearts is that the size of the atria is much smaller in pigs and the wall thickness ratio between the left and right ventricle is greater in pigs.

Differences in the vasculature include the diameter of the great vessels, such that the diameter of the ascending aorta and main pulmonary artery is proportionally smaller. Pigs also have a shorter ascending aorta, and the pig aorta has only two cerebral branches where humans have three. Pigs also have a shorter superior vena cava (SVC), so the azygous vein enters the SVC closer to the superior cavoatrial junction. Furthermore, the superior and inferior vena cavae enter the RA almost at a right angle in the pig, whereas they enter the RA in a straight line in humans. Pigs have two pulmonary veins entering the left atrium versus four in humans. Finally, the pig hemiazygous vein enters directly into the coronary sinus anterior to the left pulmonary veins, whereas this coronary sinus/hemiazygous vein branch point does not exist in humans. Many of these differences must be considered both during heart procurement as well as during transplantation of the pig heart into a human.

Although differences in the coronary circulation of pigs and humans exist, they do not impact the transplant procedure. However, these differences are important to consider after xenotransplantation if procedures such as cardiac catheterization are to be performed. Specifically, the right and left coronary arteries exit the aortic root at a smaller angle in pigs versus humans, and the left anterior descending artery in the pig does not overlie the left ventricular apex as it does in humans but shifts to the right.

Pig heart valves have been successfully transplanted into humans for >50 years because of their extensive anatomical and physiological similarities with human valves. The tricuspid and mitral valves of the pig are quite similar to those of humans with regard to size, leaflets, and chordae tendinae configuration. The pig aortic valve is slightly different in size, geometry, and fibrous continuity. Hemodynamic parameters are similar between adult pigs and humans when weight matched (173, 175).

Electrocardiogram (ECG) parameters in pigs and humans are different, such that pigs have a shorter PR interval of 50–120 ms (vs. 120–200 ms in humans), QRS of 70–90 ms (vs. 80–120 ms in humans), and QT of 260–380 ms (vs. <400 to 440 ms in humans), which may be attributed to anatomical differences in the sinoatrial node, atrioventricular node, bundle branches, and Purkinje fiber network, as well as the increased number of cholinergic and adrenergic nerves present in pigs.

Fundamental aspects of cardiac biochemistry are conserved across different species, and differences between pigs and humans identified in nucleotide metabolism have not been shown to affect cardiac function according to in vivo and clinical experience (176). However, metabolic differences between pig and human hearts have been identified such that pig hearts may be more susceptible to ischemic damage than human hearts, requiring more effective cardioprotective strategies during pig heart procurement for xenotransplantation (143, 177).

### 5.2. Kidney

Pig kidneys are similar in structure, physiology (e.g., glomerular filtration rate and total kidney blood flow), and relative size to human kidneys. However, differences between swine and human muscular layers of the genitourinary system may lead to mechanical issues in urinary flow after pig-to-human renal xenograft transplantation. Multiple groups have identified hydronephrosis or ureteral dilation leading to acute kidney injury in long-term surviving NHP recipients of renal xenografts (119, 147). Although the underlying cause is not definitively known (ureteral dilation and resulting hydronephrosis may be related to either ureterovesical stenosis or, conversely, ureteral reflux), these ureteral issues are seen at increased frequency in pig-to-NHP transplantation compared with allotransplantation and merit careful consideration and close observation in first-in-human clinical trials (K. Yamada, unpublished observations).

Physiological compatibility of the porcine kidney and the primate host is equally important to the success of pig-to-primate xenotransplantation. Fortunately, previous pig-to-NHP transplant studies suggest that, in the absence of an immune response, a transplanted pig kidney will function adequately in a human (178).

The mammalian kidney has three major functions: *1*) excretory (nitrogenous waste), *2*) regulatory (electrolyte and fluid balance), and *3*) synthetic [production of the hormones erythropoietin (EPO), renin, and 1,25-dihydroxycholecalciferol]. Renal nitrogen excretion is an essential function of the kidney, and kidney function is assessed by measuring the concentration of blood urea nitrogen (BUN). In multiple studies of pig-to-NHP renal xenotransplantation, porcine xenografts maintain stable BUN levels (134, 159, 179). The excretory function of the kidney is also commonly approximated by the serum concentration of creatinine, a by-product of muscle metabolism that is excreted unchanged by the kidneys. Serum creatinine is maintained at similar levels in swine and primates, and studies of pig-to-NHP renal xenotransplantation demonstrate maintenance of normal serum creatinine with well-functioning porcine xenografts (124, 128, 133, 180).

Regulation of electrolytes and fluid homeostasis are other important roles played by the mammalian kidney. Despite modest physiological differences between swine and human electrolyte concentrations, multiple groups have shown that sodium, potassium, and chloride remain within normal limits after pig-to-NHP xenograft transplantation (178, 181). Notably, there are significant differences between swine and primate calcium and phosphorus serum concentrations: swine phosphorus and calcium levels are higher (8.0 mg/dL and 11.0 mg/dL, respectively) than primate phosphorus and calcium (4.0 mg/dL and 9.0 mg/dL, respectively) (182). However, after pig-to-NHP renal xenograft transplantation, phosphorus levels are maintained within the low-normal range for primates, whereas calcium levels approximate high-normal levels for swine (133, 183). Moreover, preliminary unpublished results in 10 GE pigs suggest that these higher levels of serum calcium may suppress parathyroid hormone (PTH) secretion from the parathyroid glands, as significantly lower levels of PTH have been seen in baboon recipients after pig renal xenograft transplant (K. Yamada, unpublished observations).

The renin-angiotensin-aldosterone system (RAAS) is a critical regulator of renal, cardiac, and vascular physiology and has multiple functions including the regulation of blood pressure, kidney function, salt and water homeostasis, and inflammation and immune responses. Renin (produced by the kidney) cleaves angiotensinogen (produced by the liver) to form angiotensin I, which is further converted to angiotensin II (ANG II) by angiotensin-converting enzyme (184). Discrepancies have been found between pig and human RAAS such that pig renin cannot cleave human angiotensinogen (185, 186). It has also been demonstrated that baboons with pig kidney grafts have reduced circulating ANG II levels and higher plasma angiotensinogen, suggesting that pig renin may also not efficiently cleave baboon angiotensinogen and that baboons with pig kidneys may have an impaired ability to elicit a robust response to hypotensive and hypovolemic episodes (187). Nonetheless, NHPs with well-functioning pig kidneys have relatively normal fluid balance and maintain body weight but may need to drink water even when they do not feel thirsty if problems arise.

Additional potential compatibility issues may involve EPO and vitamin D metabolism. EPO is produced by the kidney in response (indirectly) to low hemoglobin levels (direct trigger is hypoxia). EPO has an ∼82% amino acid similarity between pig and human. Experimental studies revealed that after life-supporting pig-to-NHP renal xenotransplantation, long-term surviving recipients not supplemented with human EPO gradually developed anemia (117, 133, 169, 188). This may be due to several factors, including *1*) a possible lower physiological trigger for EPO production in porcine kidney due to lower normal hemoglobin concentration, *2*) reduced binding affinity of porcine EPO for human EPO receptor, *3*) myelosuppression due to immunosuppressive medications, and *4*) anemia of chronic disease due to chronic low-grade inflammation associated with a renal xenograft. Finally, it remains unknown whether the porcine kidney can produce activated vitamin D (1,25-dihydroxycholecalciferol) in baboon hosts.

In summary, the available data from pig-to-NHP kidney transplantation studies indicate normal BUN, serum creatinine, and serum electrolytes (except for a trend toward increased calcium levels). Although incompatibilities have been seen between pig and primate RAAS and EPO production, they can be resolved with hydration and human EPO supplementation, respectively.

## 6. ZOONOSES

An additional risk that must be mitigated as xenotransplantation enters clinical studies is the potential for zoonoses, or the transmission of pathogens from pigs to humans. Therefore, pigs used for xenotransplantation must be raised in designated pathogen-free facilities. Additionally, pigs must be screened for a panel of bacteria and viruses that could be zoonotic to humans, including herpes virus-gamma, swine influenza virus, porcine cytomegalovirus (pCMV), hepatitis E, and PERV.

Unlike most pig pathogens, PERVs are integrated in the genome of all pigs and thus cannot be eliminated by medication, vaccination, weaning, or embryo transfer. Moreover, PERVs are present in the germ line of all pigs and cannot be eliminated by genetic selection (189). All pigs contain PERV-A and PERV-B, but some do not have PERV-C. As PERVs are expressed in organs for xenotransplantation (190), the potential exists for zoonotic transmission.

Although certain primary human cell types including endothelial cells have been infected in vitro, viral replication was not seen (191). Only one case of productive PERV infection (characterized by viral replication) of human cells has been reported, and this was demonstrated in vitro during long-term (>6 mo) coculture of a pig cell line (PK15, which contains all 3 PERV subtypes: A, B, and C) with human embryonic kidney (HEK 293) cells. In this single case, a PERV-A/C recombinant virus was produced and transmitted to the transformed, immortalized HEK 293 cells (192), likely because of the permissiveness of these cells due to the absence of viral restriction factor APOBEC3 (apolipoprotein B mRNA editing enzyme, catalytic polypeptide-like 3G), which is an effective inhibitor of PERV. Accordingly, selection of pigs lacking PERV-C offers one approach to generating pigs for xenotransplantation, as the PERV-A/C recombination event would not be possible in cells from these pigs, thus limiting the risk of productive infection of the human recipient.

Interestingly, during breeding of Revivicor’s *Gal* KO line of pigs over multiple generations to maintain hybrid vigor, wild-type pigs were identified that were completely devoid of PERV-C. The finding of a PERV-C-negative wild-type pig line created the opportunity to breed lines of *Gal* KO pigs that would be deficient in PERV-C genomes and unable to produce potential PERV-A/C recombinants, thereby limiting the risk of infecting human cells. Over a period of 4 years, the *Gal* KO herd was bred so all *Gal* KO pigs were PERV-C free. PERV-C-free *Gal* KO cell lines were used for subsequent further genetic modifications leading up to the PERV-C-free 10 GE product pig.eGenesis used an alternative approach to generate pigs for xenotransplantation, which was to inactivate the 59 PERV elements in the genome of their pig line (171). Although this approach may reduce concerns of PERV transmission in humans undergoing porcine organ xenotransplantation, Revivicor’s approach, which is to use a pig line naturally without PERV-C, serves the same purpose.

Notably, PERVs have never been shown to be transmitted to humans or NHPs in vivo after xenotransplantation or to NHPs after experimental infection (193, 194).

## 7. INDUCTION OF TOLERANCE

Despite evidence that genetically engineered porcine xenografts, including the 10 GE porcine xenoheart and xenokidney, are not hyperacutely rejected in appropriately selected human patients (discussed below) as well as evidence extrapolated from decades of NHP data that these grafts may be life sustaining for >6 mo (138, 159, 169, 172, 195), additional strategies are under investigation to potentially improve upon these results and to promote long-term survival of heart and kidney xenografts with reduced or even eliminated maintenance immunosuppression.

Additional genetic engineering may continue to optimize source pig organs for xenotransplantation; however, concerns remain about long-term rejection-free xenograft survival given the overwhelming number of possible xenoantigens that may activate adaptive responses and antibody-mediated rejection in interspecies transplantation. Although major targets of preformed antibodies may be progressively eliminated with new iterations of product pigs, there are thousands of porcine proteins that are slightly different from their human equivalents, and it may not be possible to eliminate all sources of species incompatibility. In vitro and in vivo pig-to-NHP studies have shown greater adaptive (T and B cell) immunological responses (196), as well as increased innate responses (197) in xenotransplantation versus allotransplantation. These heightened immunological responses indicate that, like allograft recipients, xenograft recipients will likely require lifelong maintenance immunosuppression, which carries increased risks of malignancy and infectious disease. Tolerance of the transplanted graft by the recipient immune system may prove to be a critical adjunctive strategy to enable long-term xenograft survival and reduce, or even eliminate, the need for lifelong immunosuppression and associated complications.

Transplantation tolerance is realized when the recipient’s immune system does not attack the transplanted organ even in the absence of immunosuppression. In broad terms, tolerance may be induced experimentally through a variety of approaches, including hematopoietic stem cell (HSC) transplantation to achieve mixed hematopoietic cell chimerism, thymus transplantation, regulatory T-cell infusions (not discussed here), and even pharmacologically, through administration of specific immunosuppressive medications.

Although tolerance induction through mixed chimerism has been shown to allow immunosuppression-free renal allograft survival in multiple clinical studies (198–201), this approach has been more challenging to adapt across xenogeneic barriers because porcine cells are rapidly destroyed by the human immune system. Thymus cotransplantation, on the other hand, is less studied in human allogeneic transplantation; it is a particularly promising strategy for the induction of tolerance in porcine xenograft transplantation. This section briefly highlights progress and remaining challenges in the application of mixed chimerism for tolerance induction across xenogeneic barriers and then focuses on thymus cotransplantation.

### 7.1. Mixed Hematopoietic Cell Chimerism

Mixed chimerism is the coexistence of both donor and recipient hematopoietic cells. Induction of mixed chimerism-based tolerance involves nonmyeloablative conditioning followed by HSC transplantation. The mechanisms of tolerance induction after HSC transplantation are incompletely understood but initially involve suppression of donor-reactive T cells, which leads to peripheral deletion of donor-specific T cells, and ultimately intrathymic deletion of donor-reactive T cells (202). Clinical studies have demonstrated mixed chimerism-based tolerance in human kidney recipients after allotransplantation from HLA-mismatched living donors, supporting this approach in xenotransplantation (198–201).

Early studies utilizing HSC transplantation after nonmyeloablative conditioning to achieve mixed chimerism across xenogeneic barriers were encouraging: chimerism was successfully induced in rat-to-mouse transplantation (203), and tolerance with disappearance of anti-pig antibodies was achieved in pig-to-mouse models (204, 205). However, chimerism, and corresponding induction of tolerance, has proven difficult to achieve in more clinically relevant pig-to-NHP models. The central obstacle to establishment of bone marrow (BM) engraftment and durable chimerism is the rapid consumption of porcine HSCs through a combination of innate and adaptive immune mechanisms. Investigators have demonstrated that *Gal* KO porcine HSCs are eliminated from the peripheral blood within 24–48 h after infusion (206, 207).

Strategies to evade this rapid consumption of porcine HSCs have made measurable progress. Porcine HSCs expressing the human macrophage inhibitory protein hCD47 (introduced in sects. 4.9 and 4.10) have led to prolonged survival of cotransplanted porcine skin grafts in a pig-to-baboon model (157). Additionally, rather than infusing HSCs into the circulation where they are exposed to the full arsenal of innate and adaptive immunological armaments, injecting porcine HSCs directly into the BM, a strategy referred to as intrabone BM transplantation, developed by K. Yamada’s laboratory, demonstrated prolonged peripheral blood chimerism from hours to weeks and promoted BM engraftment (208). Combining these two strategies, using hCD47 transgenic porcine HSCs and intrabone BM transplantation, may have synergistic effects, prolonging porcine HSC chimerism for >60 days in peripheral blood, establishing consistent BM engraftment, and promoting survival of cotransplanted porcine lung xenograft in baboon recipients (158). Despite this improvement, mixed chimerism strategies have not achieved long-term solid organ xenograft survivals, and hematopoietic stem cell transplantation across xenogeneic barriers has not yet realized the promise of tolerance seen in clinical combined BM and kidney allograft transplantation.

### 7.2. Thymus Cotransplantation

Thymus cotransplantation, in contrast to mixed chimerism, is the only tolerance strategy that has achieved long-term xenograft survival (128, 159, 195). For reasons that are detailed in this section, thymus cotransplantation may be particularly well suited for xenotransplantation.

The thymus is the site of T-cell development and maturation, where autoreactive T cells are deleted through a variety of mechanisms, including both positive and negative selection by thymic epithelial cells (209). Since recognition of the role of the thymus in distinguishing self from nonself, thymus transplantation or thymic tissue transfer has been investigated for tolerance induction; conceptually, replacing recipient thymus with donor thymus could lead to central tolerance of cotransplanted donor-derived organs. Enthusiasm for this approach in xenotransplantation was fueled by early studies in pig-to-rodent transplantation, which demonstrated that transplantation of porcine thymic tissue led to the development of mature T cells that were tolerant of cotransplanted porcine thymic skin grafts (210). However, attempts to translate these promising findings from small- to large-animal allogeneic transplant models initially failed: nonvascularized, ischemic thymic tissue was quickly rejected before it could engraft and participate in tolerance induction (211).

**FIGURE 21.**
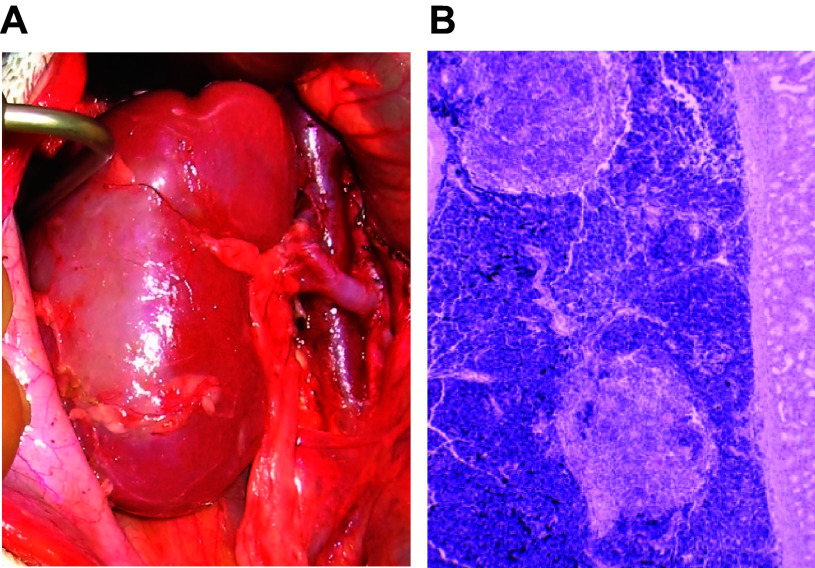
Transplantation of thymic tissue as a composite thymus and kidney. *A*: pig thymokidney in baboon soon after xenotransplantation. *B*: histology of a pig thymokidney prepared 8 wk before procurement. Image by Kazuhiko Yamada.

To enable the transfer of thymic tissue in pigs without rejection, Yamada and colleagues developed two strategies to transplant thymic tissue as a vascularized graft: *1*) transplantation of thymic tissue as a composite thymus and kidney (thymokidney) graft, where donor thymus tissue is morcellated and prevascularized under the donor renal capsule before transplantation (FIGURE 21) (212), and *2*) transplantation of vascularized thymic lobe, where the donor thymus is removed and anastomosed to the recipient’s aorta and inferior vena cava in the manner of solid organ transplantation (213). These techniques enabled thymic tissue to survive long enough to participate in T-cell development, inducing tolerance of cotransplanted kidneys across allogeneic barriers in pig-to-pig transplantation (214–216) and also inducing tolerance of cotransplanted cardiac grafts across allogeneic barriers (217). Importantly, these studies demonstrated thymopoiesis in graft thymus as well as donor-specific unresponsiveness by mixed lymphocyte reaction assays.

Demonstration of tolerance induction across allogeneic barriers using vascularized thymic grafts laid the foundation for subsequent transplantation experiments across xenogeneic barriers. At the time these techniques were initially employed in the late 1990s and early 2000s, pig-to-NHP renal xenograft survival was measured in hours and days. Greater immunological hurdles in pig-to-primate transplantation (see sect. 2) had prevented long-term graft survival despite potent (and morbid) immunosuppression regimens. While vascularized thymic grafts enabled long-term immunosuppression-free graft survival in pig-to-pig transplantation, the primary goal with tolerance approaches as they were applied across xenogeneic barriers was to prolong graft survival. In fact, thymus cotransplantation proved to be critical adjunctive therapy: utilizing single-gene modified *Gal* KO donor pigs, Yamada and colleagues (128) demonstrated that thymus cotransplantation prolonged survival of porcine renal xenografts from 29 days to >80 days in baboon recipients. Since then, additional refinements in immunosuppression (notably, addition of CTLA-4 Ig) have enabled porcine renal xenograft survival to >6 months (195), with evidence of thymopoiesis and donor-specific unresponsiveness (128).

Identification of CD80 expression on glomerular podocytes in thymokidney transplant (TKT) recipients with severe proteinuria presented another opportunity for targeted therapeutic intervention. It has been shown that CTLA-4 Ig binding to CD80 inhibits activation of CD80-expressing cells (218). Through a similar mechanism, CTLA-4 Ig improved proteinuria in *Gal* KO TKT recipients (195). Three baboon *Gal* KO TKT recipients received CTLA-4 Ig weekly starting 2 wk after transplant, and a marked reduction in proteinuria was observed with the first demonstration of long-term recipient survivals, including one recipient that survived up to 193 days (mean survival was 125 days). In contrast, four baboon *Gal* KO TKT recipients that did not receive CTLA-4 Ig developed severe proteinuria, modest glomerulopathy, high levels of urinary CD80, and documented CD80 expression on glomerular podocytes. Each recipient that did not receive CTLA-4 Ig required euthanasia before posttransplant *day 60* (mean survival was 48 days).

Together, these results suggest that anti-CD80 targeted therapy using CTLA-4 Ig can control proteinuria after *Gal* KO TKT (195). Notably, long-term acceptors of thymokidneys had pig-specific unresponsiveness in vitro as well as development of naive host T cells (CD3/CD4/CD31/CD45RAhigh) in peripheral blood of recipient baboons after native thymectomy, demonstrating pig thymic function. There was no evidence (clinical or histological) of xenograft rejection and no evidence of graft-versus-host disease.

Modifications to induction immunosuppression, including administration of rituximab (anti-CD20) to delay and CTLA-4 Ig to control proteinuria, addressed important limitations of the pig-to-baboon xenotransplant model. Combined with thymic tolerance with vascularized thymic grafts, durable survival with donor-specific immunological unresponsiveness and normal serum creatinine values have been demonstrated. In the last 5 years, Yamada and colleagues have performed six *Gal* KO pig-to-baboon TKTs using this combined immunomodulation and targeted immunosuppression regimen. Although two recipients were lost early because of complications within the first 5 wk (1 recipient lost because of line infection and another lost because of anesthetic complication), three of the four remaining recipients survived >170 days, with one animal surviving >212 days (K. Yamada unpublished observations).

Although much progress has been made in the last two decades with the creation of genetically modified source pigs, facilitating long-term survival both of xenograft kidney and of xenograft heart in pig-to-NHP models without thymus cotransplantation (138, 169, 172), adjunctive tolerance strategies remain essential for long-term graft survival of single-gene modified pigs. Indeed, there are no published studies of long-term renal xenograft survivals using single-gene modified donor pig organs without thymus cotransplantation.

Although the primary goal of thymus cotransplantation had been to prolong *Gal* KO graft survival in conjunction with immunosuppression, the availability of source pigs with multiple genetic modifications, including the 10 GE product pig (see sect. 4.14), has encouraged investigators to broaden these goals. Thymus cotransplantation has been demonstrated to enable immunosuppression-free renal allograft survival in pig-to-pig transplantation and to prolong renal xenograft survival in pig-to-NHP transplantation. Additionally, a landmark case of concurrent heart and thymus tissue allotransplantation from a single donor was conducted at Duke University in a 6-mo-old baby who had both heart failure and a T-cell deficiency (219). Approximately 6 mo after transplant, the thymus tissue was generating functional T cells. At 2 yr old, the recipient is thriving and receiving only one immunosuppression medication (220). The next steps are to achieve immunosuppression-free survival across xenogeneic barriers, translate these successes in renal xenograft transplantation to heart xenograft transplantation, and, ultimately, bring these tolerance strategies to the clinic. As detailed in sect. 9, *Gal* KO thymokidneys have been used successfully in short-term decedent studies and have recently demonstrated extended thymokidney graft survivals (up to 61 days) in the decedent model (221, 222).

## 8. TRANSLATION OF NHP RESULTS TO HUMANS

As discussed throughout this review, two decades of xenotransplantation research in NHPs has provided ample evidence that GE porcine grafts can support human xenotransplantation. However, this section summarizes key limitations of the NHP xenotransplantation model to be considered when translating NHP xenotransplantation study results to humans and designing human clinical studies (223).

NHP xenotransplantation results have been quite variable between studies and even between animals within a study (e.g., NHP survival times, graft longevity, rejection type, incidence of infection, necropsy, and histology findings). Variable results can be attributed to differences in the genetics of the source pig used, immunosuppression regimens, NHP selection protocols (e.g., level of preformed anti-donor antibodies), and the NHP species used as recipients (e.g., baboons vs. macaques). One notable difference between macaques and baboons is that macaques do not express IgG_3_, whereas baboons (and humans) express all four IgG subclasses (IgG_1–4_). Baboons are therefore considered a more stringent xenotransplantation model versus macaques (224).

Even with controlled procedures and consistent animal selection, the NHP animal model itself can impact the observed adverse events, durability of the xenoorgan, overall survival time of the recipient, and ultimately the translatability of data obtained to humans. For example, maintaining NHPs on immunosuppression for extended durations is much more difficult than caring for a patient in a clinical setting, and many of the immunosuppressive drugs have different pharmacodynamic activity and related toxicities in NHPs versus humans. Many supportive interventions used in human clinical care provided in sophisticated hospital facilities are not commonly available for NHP research in an animal facility. Plasmapheresis, blood component therapy, and surveillance graft biopsies are frequently used for human allotransplant recipients but are limited in NHPs by their availability as well as animal welfare considerations. NHP management is generally more challenging, as NHPs cannot communicate symptoms, may be aggressive, and are housed multiple animals per room, leaving them susceptible to viral and bacterial infections. Many straightforward procedures conducted for humans, such as phlebotomy and ultrasound evaluation, require deep sedation or even general anesthesia for NHPs. Additionally, to match the NHP recipient organ size, the source pigs must be young. Immediate posttransplant graft failure due to nonimmunological causes is more common with younger donors, as those organs are more susceptible to procedural damage and ischemia-reperfusion injury.

As related to zoonosis agents of concern, the main receptor for PERV entry is genetically deficient in baboons and macaques, making them inadequate preclinical models for demonstrating lack of cross-species transmission (225). Similarly, a wider selection of effective infectious disease surveillance and interventions are available for humans compared with NHPs.

The source pigs are engineered specifically for human, not NHP, transplantation. The 10 genetic modifications included in Revivicor’s pigs were selected after more than two decades of research, addressing various known interactions between pig organs and human recipients. Therefore, all modifications selected for the 10 GE pig for human transplantation may not be relevant or may even have detrimental consequences in the NHP transplant model. Clearly, inactivation of the porcine *α1,3GT* and *β4GalNT2* genes is beneficial, if not critical, for xenograft survival in both NHP and human recipients. On the other hand, the porcine *CMAH* gene knockout will likely be advantageous for human xenotransplantation but may be detrimental in the NHP model as discussed in sect. 4.13. Study results using NHP recipients and pig xenografts that include the *CMAH* knockout are unlikely to be directly translatable to outcomes in human recipients.

The six transgenes included in the 10 GE pig are derived from human (not NHP) DNA sequences. We know that complement and coagulation regulatory genes are to some extent species specific (i.e., at least the pig homologs present in the xenograft are not sufficient to regulate complement/coagulation in NHP recipients). However, the human complement and coagulation regulatory gene products seem to function in NHPs, as inclusion of at least one of each in the pig donor appears to provide a survival benefit in the NHP xenotransplantation model. It is possible that one or more of these gene products will demonstrate enhanced efficacy in humans versus NHP models.

Taken together, there are several factors unrelated to the function of the xenograft that could reduce NHP survival, which would not apply to human patients in clinical care. As studies in NHP models cannot precisely mimic physiological function and immune responses of these xenograft organs in humans, additional preclinical work in human decedents has been highly informative.

## 9. PHYSIOLOGICAL PERFORMANCE OF TRANSPLANTED GE PIG XENOHEARTS AND XENOKIDNEYS IN HUMAN DECEDENT MODELS

Eight xenotransplants have been performed with Revivicor GE pigs in preclinical human models using brain-dead recipients. These decedents had been declared dead by neurological criteria but still had a beating heart (226). Three xenokidney transplants were performed in the human preclinical decedent model (100, 101, 227, 228) using pig kidneys derived from Revivicor’s 10 GE pigs (described in sect. 4.14). The first two had a 3-day follow-up period, whereas the third was 7 days. Clinically relevant immunosuppression included ATG, rituximab, tacrolimus, MMF, and steroids in all three cases plus the complement inhibitor eculizumab in the latter two. Tacrolimus levels were subtherapeutic in the 3-day decedent studies but were within the therapeutic range in the 7-day study (100, 101, 227). Bilateral native nephrectomies were performed, so the 10 GE donor xenokidneys were fully life supporting. Before transplant, 10 GE donor lymphocytes demonstrated compatible flow cytometric crossmatch with prexenotransplant sera from all three decedents. There was no evidence of HAR in any case.

Porcine renal dysfunction persisted throughout the first decedent study; serum creatinine and BUN levels remained high, and neither kidney sufficiently excreted creatinine into the urine. Histological findings on *day 3* demonstrated endothelial injury with diffuse TMA and deposition of the MAC. The second decedent had no histological evidence of TMA after transplant, but the MAC was present. In the 7-day decedent study, the porcine kidneys produced urine, normalized creatinine, and improved creatinine clearance from 0 mL/min to 200 mL/min. Histological examination showed no evidence of TMA, although MAC deposition was observed on *days 5* and *7*, possibly due to subtherapeutic eculizumab levels. Measurements of arterial blood pressure, urine output, kidney clearance, electrolytes, and RAAS and PTH signaling components indicate that the porcine kidney provided physiological homeostasis within the human recipient, consistent with results observed after pig-to NHP renal xenograft transplantation discussed above (sect. 5) (228).

Two preclinical human decedent xenoheart transplants have also been performed with hearts from 10 GE source pigs using standard immunosuppression therapy plus eculizumab (99). Of note, the two heart xenografts did not use ex vivo perfusion, which was shown to be essential for pig-to-NHP transplantation. All human transgenes were expressed in the donor heart, and although the duration of both studies was 66 h, no evidence of cellular or antibody-mediated rejection was observed as assessed by conventional histology, flow cytometry, and cytotoxic crossmatch assays. One of the hearts exhibited graft dysfunction (declining LVEF and stroke volume) leading to irreversible ischemic injury, which may have been due to the lack of ex vivo perfusion and/or the size mismatch between the recipient and the donor heart, as the donor heart was smaller than expected. The second heart was well matched with the recipient size and performed well throughout the study, with stable left ventricular function.

Two additional xenokidney transplants were performed in decedent models using *Gal* KO pig kidneys that had autologous thymic tissue translocated under the kidney capsule (thymokidney; described in sect. 7.2) (221). The native kidneys remained in place. Standard immunosuppression was used, including steroids and MMF. Both transplants were monitored over a 54-h period. Flow cytometry and CDC analyses showed that one recipient had low levels of preformed xenoreactive IgM and IgG antibodies and a minimally positive CDC assay result, and the other recipient had moderate levels of preformed xenoreactive IgM and IgG antibodies and a positive CDC assay result. After each transplant, urine production from the porcine kidneys was twice that of the native kidneys, the creatinine level normalized, and the estimated glomerular filtration rate doubled. Although there was no evidence of TMA, interstitial hemorrhage, or antibody- or T cell-mediated rejection, microvascular inflammation and increased expression of genes involved in endothelial activation, IFNγ response, monocyte and macrophage activation, and natural killer cell burden were seen 54 h after reperfusion (229).

Most recently, a *Gal* KO thymokidney was transplanted into a human decedent, which was subsequently followed for 61 days (222). Whereas the shorter-duration (2–7 days) decedent studies showed a lack of HAR, this longer 2-mo study allowed monitoring of delayed xenograft responses. For the first month of the follow-up period, the pig kidney functioned normally. In the second month, urine production decreased and biopsy confirmed early AMR. Remarkably, using standard immunosuppression, the investigators were able to reverse the rejection event, and after removal of the kidney at the end of the study period the organ appeared normal upon gross examination.

In all eight decedent transplants, whether using 10 GE or *Gal* KO thymokidney donor organs, standard immunosuppression was used, including only clinically approved drugs. This was an important advance made possible through application of the human preclinical models, since most long-surviving NHP transplants have been shown to be dependent on some form of experimental CD40 blockade-based immunosuppression (described in sect. 4.2).

As the donor pigs were raised in a biosecure high-herd health barrier facility and monitored routinely against a panel of designated pathogens as described in sect. 6, no infectious agents of concern were observed in the donor animals before transplant. No recipients had microchimerism (where donor pig cells from the transplanted organ are shed into the decedent’s blood), and recipient PBMCs showed no evidence of PERV-A/B/C transmission by sensitive reverse transcriptase PCR assays or any evidence of porcine CMV infection by PCR or serological detection. These findings were important since they showed the absence of zoonosis in humans.

## 10. PHYSIOLOGICAL PERFORMANCE OF THE 10 GE PIG XENOHEART TRANSPLANTED INTO LIVING HUMANS

The first cardiac xenotransplantation using a GE source pig was performed on a 57-yr-old terminally ill patient with nonischemic cardiomyopathy who was dependent on extracorporeal membrane oxygenation (ECMO) and not a candidate for allotransplantation (230). The procedure was conducted at the University of Maryland on January 7, 2022, by Bartley Griffith and Muhammad Mohiuddin using a heart from Revivicor’s 10 GE pig (see sect. 4.14). This therapeutic intervention was conducted under an FDA expanded-access Investigational New Drug Application and approved by the hospital Institutional Review Board and Ethics Committee. The patient was refused consideration for allotransplantation or a heart pump in four regional and two national large centers. The cardiac graft and patient survived for 60 days after transplant (103).

Before transplant, the patient had acceptable levels of anti-pig antibodies compared with sensitized serum from baboons after pig xenograft rejection and low CDC against clonally matched 10 gene-edited pAECs. The immunosuppression regimen was designed to replicate that used by the investigators in their NHP cardiac transplantation model (138, 146). Induction therapy included rituximab and ATG for B-cell and T-cell depletion, respectively, complement C1 esterase inhibitor, a humanized anti-CD40 MAb (KPL-404; Kiniksa Pharmaceuticals), and MP. Maintenance immunosuppression included MMF, anti-CD40 MAb, and MP. Tacrolimus replaced MMF from *day 21* to *day 52*.

**FIGURE 22.**
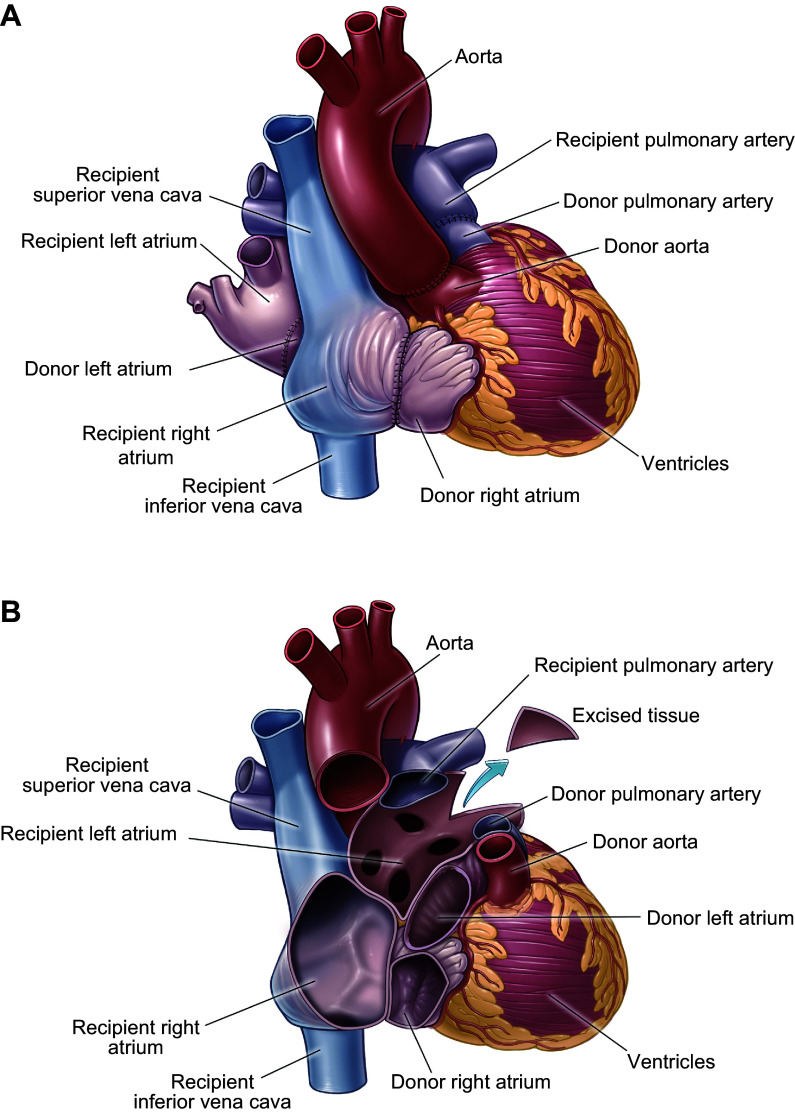
Genetically engineered pig cardiac xenograft transplanted into the first human recipient. *A*: adaptation of computed tomography (CT) 3-dimensional reconstruction imaging 7 days after xenotransplantation showing left and right atria, pulmonary artery, and aortic anastomoses. *B*: view of pig and human structures during transplantation, showing size discrepancies between pig and human atria, pulmonary artery, and aorta. A wedge of tissue from the roof of recipient’s left atrium was excised to accommodate size mismatch.

The cardiac xenograft was from a 14-mo-old GE donor pig that weighed 110 kg (the human recipient weighed 90 kg). Despite similar body weights, the heart, ascending aorta, and main pulmonary artery of the human recipient were significantly larger than those of the donor pig (see sect. 5). An adaptation of the computed tomography (CT) 3-dimensional reconstruction imaging of the GE pig cardiac xenograft in the human recipient conducted 7 days after xenotransplantation and the size mismatch between donor pig and human recipient at transplant is shown in FIGURE 22 (174). The recipient’s left atrium was over three times larger than the donor’s because of the patient’s long-standing dilated cardiomyopathy, mitral regurgitation, and the naturally small pig left atrium. This required resectional tailoring of the recipient. The recipient’s right atrium was also three times larger than that of the donor pig. The small donor atria made the donor right atrium stretch hard rightward to complete its anastomosis. The diameter of the aorta and pulmonary artery of the human recipient was about twice that of the donor pig. Regardless, the transplanted GE pig heart had no significant gradients across any of the valves or anastomoses in the recipient and generated adequate cardiac output.

**FIGURE 23.**
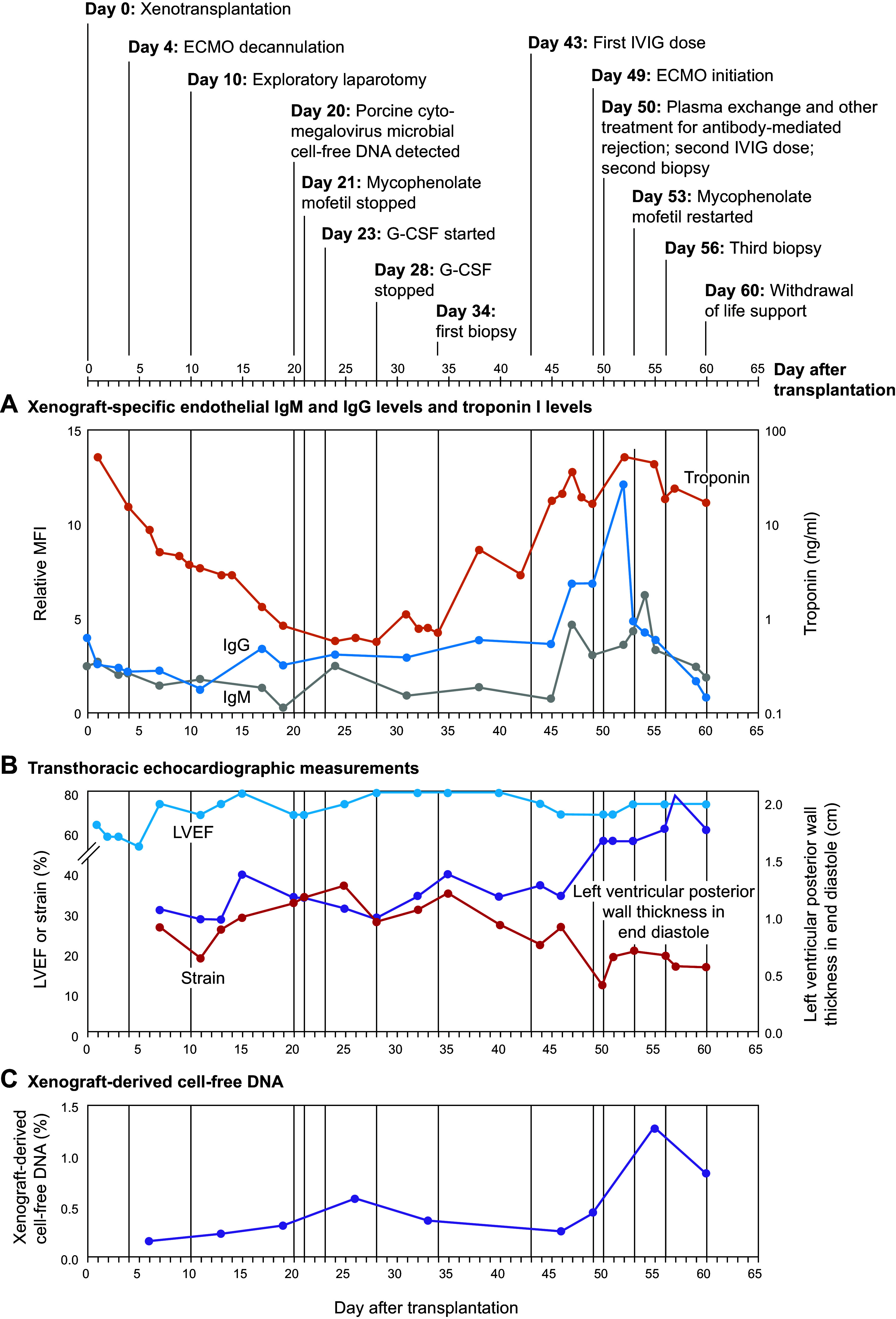
Clinical details and test results during the postoperative course. *A*: mean fluorescence intensity (MFI) values were normalized to a positive control (assigned a value of 100%). *B*: global longitudinal strain is expressed as the absolute value; strain is typically reported as a negative percentage. *C*: cell free DNA analyses of serum samples from the patient during the course of his survival. ECMO, extracorporeal membrane oxygenation; G-CSF, granulocyte colony-stimulating factor; IVIG, intravenous immune globulin; LVEF, left ventricular ejection fraction. Image from Ref. 103, reprinted with permission from Massachusetts Medical Society.

Clinical details and test results during the postoperative period are summarized in FIGURE 23. The xenograft functioned well on echocardiography and sustained cardiovascular and other organ systems functions for the first 48 days after transplant, during which time anti-pig antibody levels remained low. On *day 48*, the patient was able to leave bed and sit in a chair for the first time in >3.5 mo.

Data obtained from Swan–Ganz catheterization remained within normal limits, as did his systemic arterial pressure. Systolic and diastolic blood pressures on low-dose nicardipine were 130–170 mmHg and 40–60 mmHg, respectively. Systolic and diastolic pulmonary arterial pressures were 32–46 mmHg and 18–25 mmHg, respectively. Central venous pressure was 6–13 mmHg, cardiac output was 5.0–6.0 L/min, and stroke volume was 65–70 mL per square meter of body surface area. The sinus rhythm appeared normal between 70 and 90 beats per minute. LVEF was normal (at least 55%) throughout.

Daily 12-lead ECGs and 6-lead telemetry were also conducted throughout the postoperative period. As discussed above, normal adult pigs have shorter PR, QRS, and QT intervals relative to adult humans. The 10 GE pig cardiac xenograft transplanted into this first human patient demonstrated a prolongation of all three ECG parameters throughout the postoperative period (PR intervals of 210 ± 22 ms, QRS intervals of 145 ± 16 ms, and QT intervals of 509 ± 54 ms) and included changes in cardiac depolarization and repolarization (231). No clinically significant atrial or ventricular arrhythmias were seen despite the known proarrhythmicity of porcine hearts.

On posttransplant *day 49*, cardiac xenograft function suddenly deteriorated as indicated by the development of hypotension and acrocyanosis. Pulmonary artery catheterization showed a mixed venous oxygen saturation of 33%. Transthoracic echocardiographic measurements showed a significant increase in left and right ventricular wall thickness, reduced left ventricular chamber size, and dramatically abnormal global longitudinal strain values (232). Concomitantly, troponin I, anti-pig IgG and IgM, and porcine-derived cell-free DNA levels peaked (FIGURE 23). The patient was placed on ECMO and administered rescue therapy with plasmapheresis and antirejection medications. Because of irreversible xenograft injury, on *day 60* life support was withdrawn and the patient died (103).

Human transgene expression in the pig xenoheart was assessed by immunohistochemistry and Western blot on *day 30* after transplant and postmortem (102). Expression levels of hCD46, hCD55, hCD47, and hHO1 decreased postmortem relative to *day 30*. Expression levels of hTHBD and hEPCR (the latter of which was not detectable on *day 30*) both increased postmortem, indicating inflammation.

Although not initially apparent, the donor pig was positive for pCMV and viral RNA transcription was detected in the porcine heart after xenotransplant (102). Although the recipient was also positive for pCMV DNA, pCMV transcription or viral replication was not detected in the patient organs. Activation of latent pCMV in the xenograft may have initiated a damaging inflammatory response. There was no evidence of PERV-A, PERV-B, or PERV-C in the recipient’s PBMCs as demonstrated by PCR testing conducted 60 days after xenotransplantation.

The cause of xenograft failure appears to have been AMR. The first endomyocardial biopsy on *day 34* showed subtle endothelial injury, mild interstitial edema, and some C3d, C4d, IgG, and IgM depositions. On postoperative *day 50*, after abrupt diastolic heart failure, the endomyocardial biopsy revealed severe endothelial injury, interstitial edema with fibroblasts, red cell extravasation, C4d, IgG and IgM deposition, and degenerative changes in adjacent myocytes. Worsening endothelial injury, continued C4d, IgG, and IgM deposition, rare TMA, ischemic myocyte necrosis, and interstitial fibrosis consistent with progressive myocardial stiffness were apparent by *day 56*.

Endothelial injury and AMR may have been because of reduced immunosuppression (compared with that used in NHP studies) due to pancytopenia. Administration of commercial intravenous immune globin (IVIG) may have also contributed to the xenograft endothelial injury, as the timing of administration of the two doses correlates with sudden spikes in the level of anti-pig IgGs in the patient’s serum, and the brand of IVIG administered demonstrated strong binding to source pig AECs.

An additional cardiac xenotransplantation was performed on September 20, 2023, also at the University of Maryland Medical Center (233). A 58-yr-old man with end-stage heart failure, deemed ineligible for a traditional transplant because of his preexisting peripheral vascular disease and complications with internal bleeding, became the second patient in the world to receive a successful transplant of a genetically modified pig heart from Revivicor’s 10 GE pig. Shortly before the transplant surgery, his native heart stopped, requiring resuscitation. After the transplant, the patient was engaged in physical therapy and working toward regaining his ability to walk. However, the donor heart began to develop diastolic dysfunction and failure (similar to that seen in the first case), likely driven by AMR. Approximately 6 wk after transplant the patient died.

The cause of AMR is still under investigation and may have been due to reduced immunosuppression. The patient also received blood and blood products because of bleeding, which on later testing were found to contain significant levels of anti-pig antibodies and may have contributed to AMR. Consensus pharmaceutical and mechanical interventions will be used to mitigate AMR during future clinical xenotransplantation cases.

As noted above, the donor pig for the first human cardiac xenotransplant was positive for pCMV. A more sensitive assay was developed and used to screen this second source pig, and no pCMV was detected in the donor organ or recipient after transplant. No other porcine viruses were detected in the xenoheart or the recipient.

## 11. FUTURE CONSIDERATIONS

### 11.1. Additional Genetic Modifications

A variety of additional genetic modifications have been generated in multitransgenic pigs and tested in NHP lung, kidney, heart, liver, and pancreatic islet xenotransplantation models. TABLE 2 provides a summary of genetic modifications that have been generated by multiple groups and tested in a variety of preclinical studies. Human tissue factor protein inhibitor (*hTFPI*) and human *CD39* anticoagulant transgenes were used in combination with *Gal* KO and *hCD46* genetics, whereby the anticoagulant transgenes were under control of a pancreatic beta cell-specific promoter to generate GE islets for correction of diabetes. The addition of hTFPI helped address the instant blood-mediated inflammatory response that occurs when islets are infused into the portal vein into the liver and resulted in reduced islet destruction in the early postinfusion phase (249). Transplants in a diabetic cynomolgus macaque model with delivery of the *hCD46* transgenic islets via the portal vein demonstrated complete glucocorrection and normalization of diabetes up to 1 yr (250).

**Table 2.** Beyond the 10 GE pig: genetic modifications with potential to improve

Modification	Function	Reference
*Complement inhibitors*		
*hCD59*	Inhibits complement membrane attack complex.	(234)
*Thrombosis inhibitors*		
*hTFPI*	Inhibits tissue factor; potent anticoagulant.	(235, 236)
hvWF*	Inhibits spontaneous human platelet aggregation in porcine organs.	(237)
*Immune modulation*		
*HLA-E, HLA-G*	Anti-NK human cell activity	(238, 239)
*CIITA-DN*†	MHC class II inhibitor	(240)
*CTLA-4 Ig*	Inhibits T cell-mediated rejection.	(241, 242)
*hPDL-1*	Inhibits T cell-mediated rejection.	(243)
*Anti-inflammatory*		
*CD39*	Inhibits inflammation, apoptosis.	(244–246)
*CD73*	Blocks NK-mediated cytotoxicity.	(246, 247)
*A20*	Inhibits inflammation, apoptosis.	(248)

*Chimeric porcine *vWF* in which the native *GPIb* platelet binding domain was replaced with human sequence. †Class II transactivator, with dominant negative mutation. See glossary for abbreviations.

Lungs from transgenic pigs expressing human *HLA-E* (on the background of *Gal* KO.*hCD46*) showed a rapid loss of NK cells, increased median lung survival, reduced pulmonary vascular resistance, and decreased platelet activation and histamine elaboration in ex vivo lung perfusion studies (251). In addition, in vitro studies showed that endothelial cells derived from *Gal* KO.*hCD46.HLA-E* transgenic pigs were significantly protected against human NK cell-mediated cytotoxicity (238). To further address molecular incompatibilities specific to lung xenografts, pigs with humanized von Willebrand factor (vWF) were developed (237). By replacing a region of porcine *vWF* that encodes the glycoprotein 1b-binding site with the human cDNA ortholog, lungs and livers from such pigs demonstrated reduced platelet sequestration when perfused ex vivo with human blood. The longest life-supporting lung xenograft, from a pig with seven genetic modifications (including inactivation of *α1,3GT*, partial inactivation of *β4GalNT2*, plus human transgenes *hCD46, hTHBD, hEPCR, hCD47*, and *hHO1*) survived for 31 days in a baboon (252). It is anticipated that the addition of *HLA-E* and/or humanization of *vWF* to this 7 GE genetic background could further prolong xenolung survival.

Toward creating donor organs requiring reduced or allograft levels of immunosuppression, the Revivicor team cloned the porcine *CTLA-4 Ig* gene, under control of a constitutive *CAG* promoter, and used this vector to create *Gal* KO.*hCD46.CTLA-4 Ig* transgenic pigs (241). These pigs expressed *pCTLA-4 Ig* in blood and all organs; however, because of diminished humoral immunity, they were susceptible to recurrent infections and thus not a sustainable line for therapeutic use.

As a further endeavor to reduce immune responses in xenograft organs, Revivicor pigs were generated with a transgene designed to reduce or knock down (not knock out) porcine SLA class II. Overexpression of a dominant-negative mutant human MHC class II transactivator (*CIITA*) resulted in reduced expression of *SLA DR* and *DQ* and reduced sensitization to non-Gal antigens in *Gal* KO.*CIITA* artery patch grafts in baboons (253).

### 11.2. GalSafe Medical Products and Food for People with αGal Syndrome

Although the multiple genetic modifications described for the 10 GE pigs have shown to be critical for extended survivals of heart and kidney whole organ xenotransplants, the *Gal* KO (GalSafe) pig, which was approved by the FDA in 2020 for both consumption and medical products, may also prove an important source of medicaments for human clinical applications (254). The αGal epitope is ubiquitously present on cells, tissues, and organs of not only pigs but all food-producing mammals (e.g., beef, pork, and lamb) as well as bovine and porcine derived bioscaffolds. The lack of detectable αGal sugar on the cell surfaces of GalSafe pigs has implications for people who suffer from αGal syndrome (AGS), an allergy to red meat and other products containing mammalian-based materials, including cosmetics and medicines (255). This allergy, which was first discovered in the United States in the mid-2000s, occurs in some people after they are bitten by a lone star tick (*Amblyomma americanum*) and certain other tick species (256–259). The tick bite transmits αGal sugar molecules into the person’s body and, in some people, triggers an IgE-mediated immune response that later produces an allergic reaction after consumption of red meat or other products containing mammalian-based materials. Some people bitten by a lone star tick may display mild allergic reactions but not be diagnosed with AGS. Other people experience more severe reactions, including anaphylaxis requiring immediate medical care.

The reported prevalence of individuals (adults and children) in regions of the United States with elevated allergen-specific titers of anti-αGal IgE (i.e., allergen positive) has been reported to be in the range of 8–46%, with highest prevalence commensurate with the geographical range of the arachnid responsible for initial sensitization (260–266). Similar observations have been reported for prevalence in other regions around the world (263, 264). In 2023, the Centers for Disease Control and Prevention released figures estimating the incidence of AGS in the United States as 450,000 people (267), numbers that make this one of the top 10 allergies. Food products made from GalSafe pigs contain undetectable αGal sugar and may provide a red meat option for people with AGS. Furthermore, AGS patients may have allergic reactions to mammalian-derived materials including heparin (blood thinner), gelatin as an excipient, and therapeutic enzymes and hormones, which would be alleviated if such products were made from GalSafe pigs.

Tissue decellularization is intended to reduce immunogenicity while preserving the beneficial properties of the extracellular matrix. Many current tissue products, including heart valves, small intestinal submucosa for hernia repair, dermis, and orthopedic repair devices are mammalian derived and despite decellularization still ubiquitously express αGal (268, 269). Regardless of the decellularization process, the αGal epitope persists in those medical products and can potentially trigger an IgG- or IgE-mediated response. GalSafe pigs may also provide an alternative and safer source of porcine-based biomedical materials.

### 11.3. Potential Use of Genetically Modified Pig Heart Valves in Humans

Heart valves perform extremely sophisticated functions that can influence both survival and quality of life (270, 271). These integrated functions require multiscale structure at the molecular, cellular, and tissue levels. Initial surgical application of intracardiac insertion of heart valves in humans involved allografts and was performed in the 1960s by Donald Ross (272) and Brian Barratt-Boyes (273) with techniques that were a modification of the original technique described by Duran and Gunning (274). At that time, it was hoped that the human allograft would continue to survive, as it was being inserted in what was thought to be an immunologically privileged site and it was virtually avascular (271). This proved to not be the case, as there was definite evidence that the aortic allograft evoked an immunological reaction that was donor specific, and these valves became acellular after a period of 1–2 mo (275, 276).

Because of the limited supply of heart valves from human cadavers, porcine bioprosthetic heart valves (BPVs) were investigated for use as an alternative (277), and the Hancock porcine BPV became commercially available in 1972 (278). These valves are now cross-linked with glutaraldehyde and/or decellularized to increase stability and sterility and to reduce antigenicity. Although mechanical valves are more durable than BPVs, they do not demonstrate the physiological hemodynamics achieved with BPVs, which contributes to thrombosis requiring the recipient to receive lifelong anticoagulation (279).

Not surprisingly, as seen in homograft heart valves, xenograft BPVs induce an immunological reaction, despite glutaraldehyde cross-linking and decellularization. Several studies have provided evidence that deleterious immune responses contribute to calcification and deterioration of BPVs (280). Specifically, the αGal antigen, which is present on most commercially available BPVs, may play a role in the immune response, as patients who received these valves have an increase in anti-Gal antibody after implantation relative to patients who did not receive BPVs. Similarly, NHP recipients of wild-type pig valves developed significantly higher anti-Gal antibody levels 1 yr after implant versus those who received valves from *Gal* KO pigs (281). Furthermore, in the presence of human anti-Gal antibody, glutaraldehyde-treated pericardial tissue from wild-type pigs demonstrated significantly greater calcification after transplantation into rodents than that from *Gal* KO pigs (281).

With the link established between the αGal epitope and structural deterioration of xenogenic BPVs, bioprosthetics sourced from GalSafe pigs may have extended durability relative to those currently on the market. Although this section discusses immune responses of people with AGS to αGal-containing biomedical tissue products, these results indicate that people without diagnosed AGS may also react to a certain degree to αGal-containing biomedical materials, reducing the functional life of these products.

## 12. CONCLUSIONS

This review describes the physiological homologies of certain vital organs between pigs and humans that make xenotransplantation practical once the pigs have been appropriately gene modified and immunologically matched with human recipients. All leading schools of ethical thought, including major religions, support the use of pigs for xenotransplantation provided there are no other ways to save the patient’s life, the patient gives informed consent, and reasonable measures are undertaken to safeguard the health of others from possible xenoviruses (282, 283).

## GLOSSARY

10 GE: Genetically engineered pig with 10 genetic modifications
ACT: Activated clotting time
ADCC: Antibody-dependent cellular cytotoxicity
AGS: αGal syndrome
AMR: Antibody-mediated rejection
ANG II: Angiotensin II
APC: Antigen-presenting cell
ATG: Antithymocyte globulin
BM: Bone marrow
bp: Base pair
BUN: Blood urea nitrogen
Cas: CRISPR-associated
CDC: Complement-dependent cytotoxicity
CMAH: Cytidine monophosphate-N-acetylneuraminic acid hydroxylase
CNI: Calcineurin
CRISPR: Clustered regularly interspaced short palindromic repeat
CRP: Complement regulatory protein
CT: Computed tomography
CTLA-4: Cytotoxic T lymphocyte-associated protein 4
CVF: Cobra venom factor
DAF: Decay accelerating factor
DSB: Double-strand break
DXR: Delayed xenograft rejection
ECG: Electrocardiogram
ELISPOT: Enzyme-linked immunospot
EPCR: Extracellular protein C receptor
EPO: Erythropoietin
ES: Embryonic stem
FITC: Fluorescein isothiocyanate
G-CSF: Granulocyte colony-stimulating factor
GE: Genetically engineered
GHr: Growth hormone receptor
GLS: Global longitudinal strain
HAR: Hyperacute rejection
hCD: Human CD molecule
hCRP: Human CRP
HCT: HSC transplantation
HDR: Homology-directed repair
HEK 293: Human embryonic kidney 293
hEPCR: Human EPCR
HLA: Human leukocyte antigen
HO1: Heme oxygenase-1
HSC: Hematopoietic stem cell
hTHBD: Human THBD
hvWF: Human von Willebrand factor
IFN: Interferon
IGF-1: Insulin-like growth factor 1
IgG: Immunoglobulin G
IgM: Immunoglobulin M
indels: Insertion/deletion
IVIG: Intravenous immune globin
KO: Knockout
LVEF: Left ventricular ejection fraction
MAb: Monoclonal antibody
MAC: Membrane attack complex
MFI: Mean fluorescence intensity
MHC: Major histocompatibility complex
MLR: Mixed lymphocyte reaction
MMF: Mycophenolate mofetil
MP: Methylprednisone
mTOR: Mammalian target of rapamycin
*neoR*
: Neomycin resistance gene
Neu5Gc: N-glycolylneuraminic acid
NHEJ: Nonhomologous end joining
NHP: Nonhuman primate
NICP: Nonischemic continuous perfusion
NK: Natural killer
pAEC: Porcine aortic endothelial cell
PAP: Pulmonary arterial pressure
PBMC: Peripheral blood mononuclear cell
pCMV: Porcine cytomegalovirus
PCR: Polymerase chain reaction
PCXD: Perioperative cardiac xenograft dysfunction
PERV: Porcine endogenous retrovirus
PTH: Parathyroid hormone
pTHBD: Porcine THBD
RA: Right atrium
RAAS: Renin-angiotensin-aldosterone system
RBC: Red blood cell
RNA: Ribonucleic acid
RTE: Recent thymic emigrant
SCNT: Somatic cell nuclear transfer
sCR-1: Soluble complement receptor-1
SDa: Sialyl-dimeric antigen
SIRP-α: Signal regulatory protein alpha
SLA: Swine leukocyte antigen
SPF: Specific pathogen free
SVC: Superior vena cava
TALENS: Transcription activator-like effector nucleases
TCR: T-cell receptor
TFPI: Tissue factor protein inhibitor
THBD: Thrombomodulin
TKT: Thymokidney transplant
TMA: Thrombotic microangiopathy
TTE: Transthoracic echocardiography
vWF: Von Willebrand factor
WT: Wild type
α1,3GT: α(1,3)Galactosyltransferase
αGal: Galα1-3Galβ1-4GlcNAc-R epitope
β4GalNT2: β1,4 N-acetylgalactosaminyltransferease-2

## DISCLOSURES

L.P., D.A., W.E., and M.R. are employees of United Therapeutics. M.Y. is on the scientific advisory board of United Therapeutics. J.H.U. and U.M.B. have scientific research agreements with United Therapeutics. None of the other authors has any conflicts of interest, financial or otherwise, to disclose.

## AUTHOR CONTRIBUTIONS

D.A., K.Y., M.M., and M.R. conceived and designed research; D.A., K.Y., D.E., B.G., and M.M. performed experiments; K.Y., D.E., and M.M. analyzed data; L.P., D.A., K.Y., D.E., and M.M. interpreted results of experiments; L.P. prepared figures; L.P., M.Y., D.A., K.Y., D.E., W.E., and M.R. drafted manuscript; L.P., M.Y., D.A., K.Y., D.E., W.E., J.C.V., R.T.S., and M.R. edited and revised manuscript; L.P., M.Y., D.A., K.Y., D.E., B.G., M.M., W.E., J.C.V., R.T.S., and M.R. approved final version of manuscript.
